# Trends in Frequency of Sexual Activity and Number of Sexual Partners Among Adults Aged 18 to 44 Years in the US, 2000-2018

**DOI:** 10.1001/jamanetworkopen.2020.3833

**Published:** 2020-06-12

**Authors:** Peter Ueda, Catherine H. Mercer, Cyrus Ghaznavi, Debby Herbenick

**Affiliations:** 1 Clinical Epidemiology Division, Department of Medicine, Solna, Karolinska Institutet, Stockholm, Sweden; 2 Department of Global Health Policy, Graduate School of Medicine, University of Tokyo, Tokyo, Japan; 3 Centre for Population Research in Sexual Health and HIV, Institute for Global Health, University College London, London, United Kingdom; 4 Department of Medicine, Washington University School of Medicine, St Louis, Missouri; 5 Center for Sexual Health Promotion, School of Public Health, Indiana University, Bloomington

## Abstract

**Importance:**

Sexual relationships are important for well-being and health. Recent trends in sexual activity among US adults are unknown.

**Objectives:**

To examine trends in reported frequency of sexual activity and number of sexual partners by sex and age and the association between measures of sexual activity and sociodemographic variables.

**Design, Setting, and Participants:**

In this survey study, repeat, cross-sectional analyses of participants aged 18 to 44 years from 10 rounds of the General Social Survey (2000-2018), a US nationally representative survey, were performed for men and women separately.

**Main Outcomes and Measures:**

Sexual frequency in the past year (sexual inactivity, once or twice per year, 1-3 times per month, or weekly or more) and number of sexual partners in the past year (0, 1, 2, or ≥3 partners). The association between measures of sexual activity and sociodemographic variables were assessed using logistic regression.

**Results:**

The study population included 4291 men and 5213 women in the analysis of sexual frequency and 4372 men and 5377 women in the analysis of number of sexual partners (mean [SD] age, 31.4 [7.6] years; survey response rate, 59.5%-71.4%). Between 2000-2002 and 2016-2018, the proportion of 18- to 24-year-old individuals who reported having had no sexual activity in the past year increased among men (18.9% vs 30.9%; age-adjusted odds ratio [aOR] for trend across survey periods, 1.20; 95% CI, 1.04-1.39) but not among women (15.1% vs 19.1%; aOR for trend, 1.03; 95% CI, 0.89-1.18). Smaller absolute increases in sexual inactivity were observed among those aged 25 to 34 years for both men (7.0% vs 14.1%; aOR for trend, 1.23; 95% CI, 1.07-1.42) and women (7.0% vs 12.6%; aOR for trend, 1.17; 95% CI, 1.01-1.35) but not among those aged 35 to 44 years. The increase in sexual inactivity coincided with decreases in the proportion reporting weekly or more sexual frequency (men aged 18-24 years: 51.8% vs. 37.4%; aOR for trend, 0.88 [95% CI, 0.79-0.99]; men aged 25-34 years: 65.3% vs 50.3%; aOR for trend, 0.87 [95% CI, 0.81-0.94]; women aged 25-34 years: 66.4% vs. 54.2%; aOR for trend, 0.90 [95% CI, 0.84-0.96]) or 1 sexual partner (men aged 18-24 years: 44.2% vs. 30.0%; aOR for trend, 0.88 [95% CI, 0.80-0.98]; women aged 25-34 years: 79.6% vs 72.7%; aOR for trend, 0.91 [95% CI, 0.84-0.99]) and occurred mainly among unmarried men (unmarried men aged 18-44 years: 16.2% vs 24.4%; aOR for trend, 1.14 [95% CI, 1.04-1.25]). Among married men and women, weekly or more sexual frequency decreased (men: 71.1 % vs 57.7%; aOR for trend, 0.86 [95% CI, 0.79-0.93]; women: 69.1% vs 60.9%; aOR for trend, 0.92 [95% CI, 0.86-0.99]). Men with lower income (aOR for men with an annual income of ≥$50 000 vs $0-$9999, 0.37 [95% CI, 0.15-0.90]) and with part-time (aOR vs full-time employment, 2.08; 95% CI, 1.48-2.93) and no employment (aOR vs full-time employment, 2.08; 95% CI, 1.48-2.93) were more likely to be sexually inactive, as were men (aOR vs full-time employment, 2.94; 95% CI, 2.06-4.21) and women (aOR vs full-time employment, 2.37; 95% CI, 1.68-3.35) who were students.

**Conclusions and Relevance:**

This survey study found that from 2000 to 2018, sexual inactivity increased among US men such that approximately 1 in 3 men aged 18 to 24 years reported no sexual activity in the past year. Sexual inactivity also increased among men and women aged 25 to 34 years. These findings may have implications for public health.

## Key Points

**Question:**

Did the distribution of sexual frequency and number of sexual partners in the past year among US adults change between 2000 and 2018, and was there an association between sexual activity and sociodemographic variables?

**Findings:**

In this survey study of US adults from 2000 to 2018, sexual inactivity increased among men aged 18 to 24 years and 25 to 34 years and women aged 25 to 34 years during the study period, with the increase among men mainly occurring among unmarried individuals. Men with lower income and with part-time or no employment were more likely to be sexually inactive, as were men and women who were students.

**Meaning:**

This study found that sexual inactivity increased among US adults, predominantly younger men, between 2000 and 2018, with potential public health implications.

## Teaser

This survey study of adults aged 18 to 44 years examines trends in reported frequency of sexual activity and number of sexual partners and the association between measures of sexual activity and sociodemographic variables.

## Introduction

Sexual health and satisfaction are key components of health and well-being.^1,2,3^ Sexual relationships can positively influence life satisfaction and happiness,^4,5,6^ and sexual activity may lower heart rate and blood pressure^7,8^ while also reducing stress by promoting oxytocin release.^9^ Conversely, lower sexual activity has been associated with increased mortality^10^ and poor self-reported health,^11^ although these associations warrant careful interpretation because sexual activity and health outcomes may have common causes and healthier individuals may have more sexual activity.

Although sexual inactivity and sexual frequency have recently been subject to increased scrutiny from public health perspectives,^11,12,13,14^ uncertainty remains regarding recent trends in sexual activity among US adults. A study^15^ using nationally representative data from the General Social Survey (1972-2014) found that at 20 to 24 years of age, 6.3% of Americans born in 1965 to 1969 reported having had no sexual partners after 18 years of age. This proportion was 11.5% for those born in 1970 to 1979, 11.7% for those born in 1980 to 1989, and 15.2% for those born in 1990 to 1994. Although this study^15^ found that sexual inactivity in one’s early 20s was less common among those born in 1965 to 1969 than in subsequent generations, to our knowledge, trends in sexual inactivity and in regular sexual frequency have not been assessed using a wider range of age and sex groups and more recent data. Another study^16^ using data from the same survey estimated that US adults (aged ≥18 years) had sexual frequencies of approximately 9 fewer times per year in the early 2010s compared with the late 1990s. Because these analyses did not account for the distribution of sexual activity in the population, it is unclear whether this finding was attributable to a decreased sexual frequency among sexually active adults or whether it represented an increase in the proportion who did not have sexual activity at all. This distinction is important because the societal and public health implications of the 2 potential mechanisms differ substantially.

Using data from 18- to 44-year-old participants in the General Social Survey from 2000 to 2018, we assessed trends in categories of sexual frequency (including sexual inactivity) and number of sexual partners in the year preceding survey participation. We then examined factors associated with sexual frequency and the number of sexual partners.

## Methods

### Study Population

The General Social Survey is a nationally representative, biennial survey of US adults 18 years or older (eAppendix in the Supplement).^17^ We used data from 10 waves of the survey in 2000 to 2018. We included all participants aged 18 to 44 years who had been asked the questions regarding sexual frequency and number of sexual partners in the past year, as described below (eAppendix and eTable 1 in the Supplement). For analyses on sexual frequency, we excluded 381 individuals with unknown sexual frequency (weighted, 3.9%), and for analyses on number of sexual partners, we excluded 136 with unknown number of partners (weighted, 1.3%) (eTable 2 in the Supplement). The General Social Survey was approved by the institutional review board at the National Opinion Research Center, University of Chicago. Participants provided oral informed consent for interviews. All data were deidentified. The survey is conducted by the National Opinion Research Center at the University of Chicago, which participates in the American Association for Public Opinion Research (AAPOR) Transparency Initiative and follows best practices in survey research.

### Measures of Sexual Activity

We assessed 2 measures of sexual activity in the past year: sexual frequency and number of sexual partners. The survey included the question, “About how often did you have sex during the last 12 months?” with response options ranging from “not at all” to “more than 3 times a week” (eAppendix in the Supplement). We categorized sexual frequency in the past year into (1) sexually inactive (no sex during the past year), (2) once or twice per year, (3) 1 to 3 times per month, and (4) weekly or more. The question, “How many sex partners have you had in the last 12 months?” could be answered with choices ranging from “no partners” to “more than 100 partners” (eAppendix in the Supplement). We categorized number of sexual partners in the past year into (1) no partners, (2) 1 partner, (3) 2 partners, and (4) 3 or more partners. The rationale for the categories used is provided in the eAppendix in the Supplement.

### Statistical Analysis

Analyses were performed in Stata, version 15.0 (StataCorp) and accounted for the stratification, clustering, and weighting of the samples. Analyses were performed separately by sex because of differences in the experience and reporting of sexual behaviors^18^ and the meaning and interpretation that shape these behaviors.^19^

First, we used the 2016-2018 survey to assess the proportion of the population in each category of sexual frequency and number of sexual partners in the total age range and by age group (18-24, 25-34, and 35-44 years, as in previous studies^12,13,18^), using the χ^2^ test to assess differences in proportions between sexes. Second, we estimated these proportions in all surveys, grouped pairwise to increase statistical power (2000-2002, 2004-2006, 2008-2010, 2012-2014, and 2016-2018), in the total age range, by age group, and by marital status. All reported percentages are weighted. We applied logistic regression to assess trends over time for each category of sexual frequency and number of sexual partners by calculating age-adjusted odds ratios (aORs) using the investigated category of sexual frequency or number of partners as the dependent variable and age and survey period (survey pairs as described above) as independent variables.

We assessed the association between sociodemographic and behavioral variables and sexual inactivity, weekly sex or more, no sexual partners, and ≥3 sexual partners in the past year. We focused on these measures of sexual activity because they constitute the extremes of the categories used in our analyses and have been used in previous studies.^12,14,20^ We calculated aORs using the investigated measure of sexual activity as the dependent variable and age, survey period, and the variable of interest as the independent variables. Definitions of and the rationale for analyzing the sociodemographic and behavioral variables are given in eTable 3 in the Supplement.

Because the pairwise grouping of surveys may have obscured trends in the investigated measures of sexual activity, we performed additional analyses in which we analyzed each survey year separately. Because we found statistically significant trends in sexual inactivity, weekly or more sexual activity, and having no sexual partner among men, we performed post hoc analyses in which we assessed these trends in sociodemographic subgroups of men. To examine whether changes in the distribution of sociodemographic characteristics could explain the observed trends, we also assessed the trends using multivariable logistic regression models adjusted for the sociodemographic variables that were available during the entire study period (eTable 3 in the Supplement). Finally, we assessed the distribution of sexual frequency and number of sexual partners among participants identifying as gay, lesbian, or bisexual.

## Results

The study population included 4291 men and 5213 women in the analysis of sexual frequency and 4372 men and 5377 women in the analysis of number of sexual partners (mean [SD] age, 31.4 [7.6] years). Survey response rates ranged from 59.5% to 71.4% (eAppendix in the Supplement). Sample characteristics are given in eTable 4 in the Supplement.

### Measures of Sexual Activity in 2016-2018

**Figure 1.**
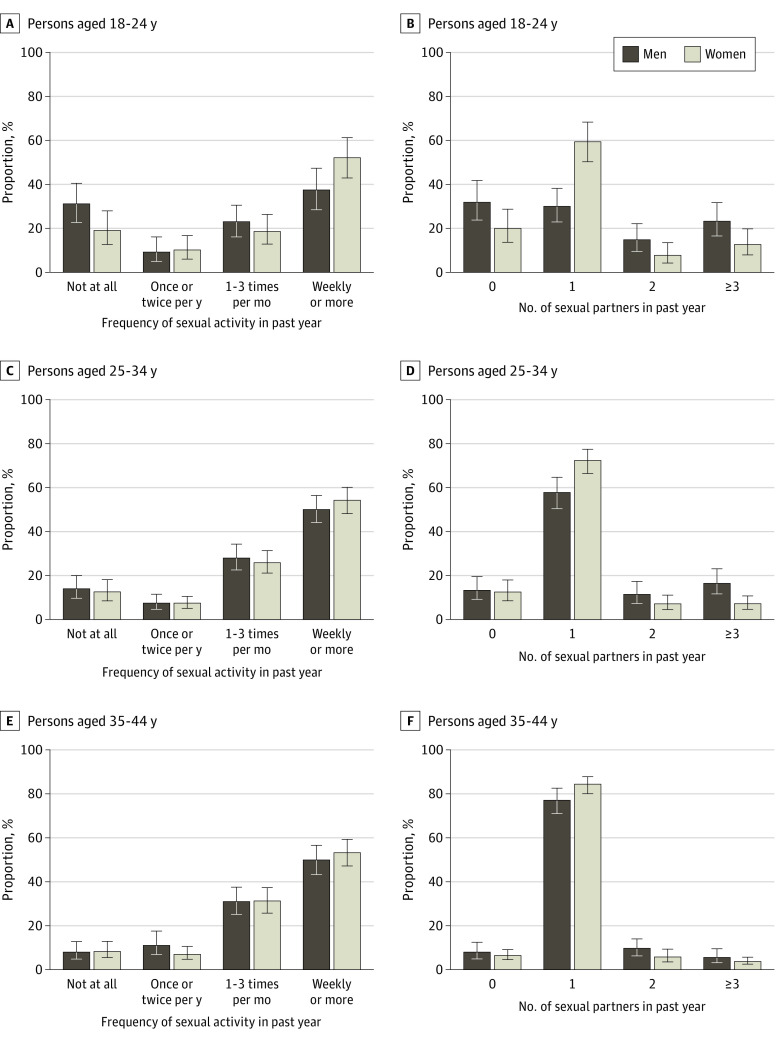
Distribution of Frequency of Sexual Activity and Number of Sexual Partners in the Past Year Among US Men and Women in the General Social Survey, 2016-2018, by Age Group Error bars represent 95% CIs. The total age range is shown in eFigure 1 in the Supplement.

Figure 1 and eTable 5 and eFigure 1 in the Supplement give the proportion of US men and women by category of sexual frequency and number of sexual partners in 2016-2018. Overall, most men and women reported having had weekly or more sexual activity and 1 sexual partner in the past year, with these percentages increasing with age. More men than women reported having no sexual partner (16.4% vs 12.0%; *P* = .04) and 3 or more partners (14.5% vs 7.1%; *P* < .001), whereas fewer men reported weekly or more sexual activity (46.7% vs 53.3%; *P* = .02) and 1 sexual partner (57.5% vs 74.2%; *P* < .001). Differences between the sexes were most pronounced among those aged 18 to 24 and 25 to 34 years.

### Trends in Sexual Frequency and Number of Sexual Partners

**Figure 2.**
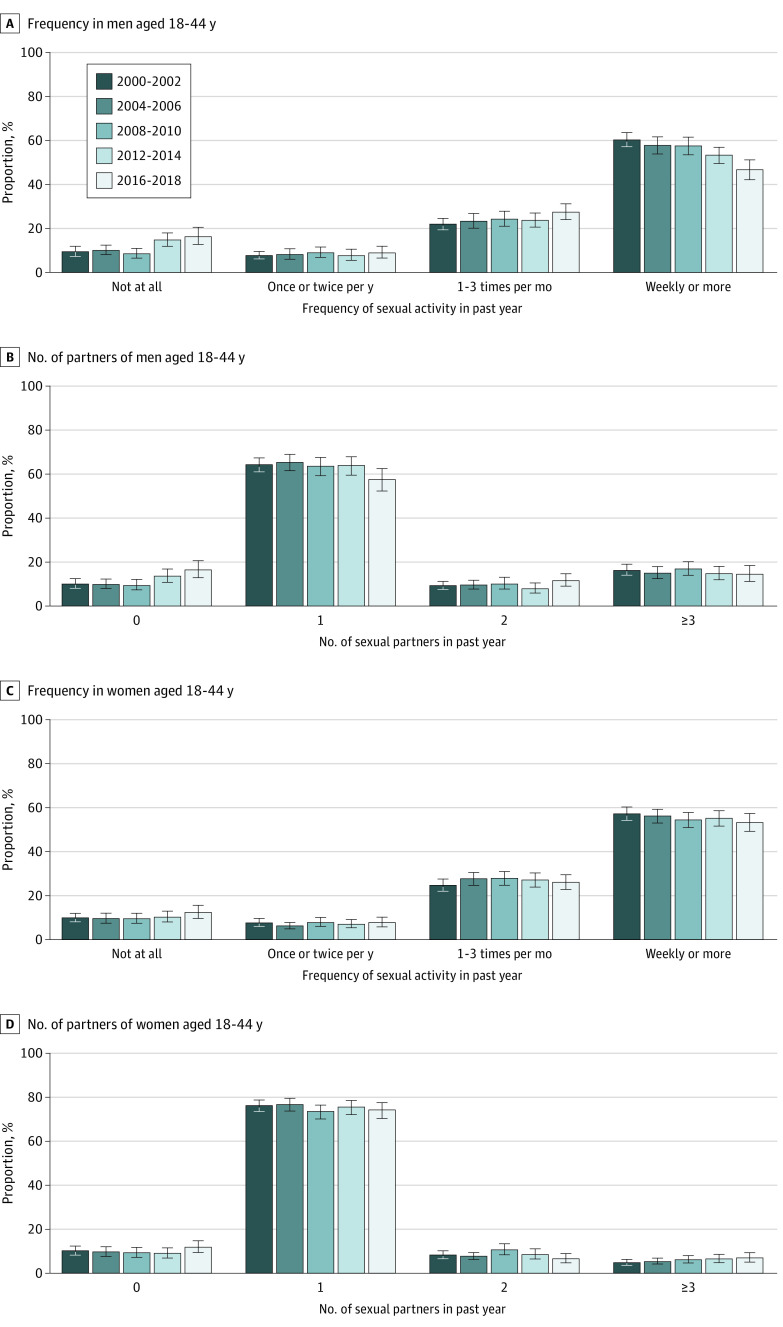
Trends in Frequency of Sexual Activity and Number of Sexual Partners in the Past Year Among US Men and Women Aged 18 to 44 Years Error bars represent 95% CIs.

**Figure 3.**
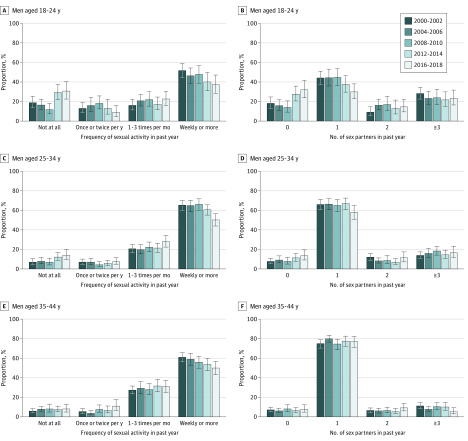
Trends in Frequency of Sexual Activity and Number of Sexual Partners in the Past Year Among US Men by Age Group Error bars represent 95% CIs.

In the total age range, sexual inactivity among men increased from 9.5% in 2000-2002 to 16.5% in 2016-2018 (aOR for trend across survey periods 1.18; 95% CI, 1.08-1.29), with most of the increase occurring between 2008-2010 and 2012-2014 (Figure 2 and Figure 3 and eFigure 2, eTable 6, and eTable 7 in the Supplement). Decreases were observed in the proportion reporting weekly or more sexual activity (60.4% in 2000-2002 vs 46.7% in 2016-2018; aOR, 0.89; 95% CI, 0.84-0.93) and those reporting 1 sexual partner (64.3% vs 57.5%; aOR, 0.95; 95% CI, 0.90-1.00).

The absolute increase in sexual inactivity was most pronounced among men aged 18 to 24 years (18.9% vs 30.9%; aOR, 1.20; 95% CI, 1.04-1.39). In this age group, the proportions of those reporting weekly or more sexual activity (51.8% vs 37.4%; aOR, 0.88; 95% CI, 0.79-0.99) and those reporting 1 sexual partner (44.2% vs 30.0%; aOR, 0.88; 95% CI, 0.80-0.98) decreased. Among men aged 25 to 34 years, sexual inactivity doubled from 7.0% to 14.1% (aOR, 1.23; 95% CI, 1.07-1.42), and weekly or more sexual activity decreased from 65.3% to 50.3% (aOR, 0.87; 95% CI, 0.81-0.94). In men aged 35 to 44 years, sexual inactivity was largely unchanged during the study period, whereas a sexual frequency of 1 to 3 times per month increased slightly and weekly or more sexual activity decreased from 61.1% to 49.9%; aOR, 0.89; 95% CI, 0.83-0.96) (Figure 3 and eFigure 2, eTable 6, and eTable 7 in the Supplement).

**Figure 4.**
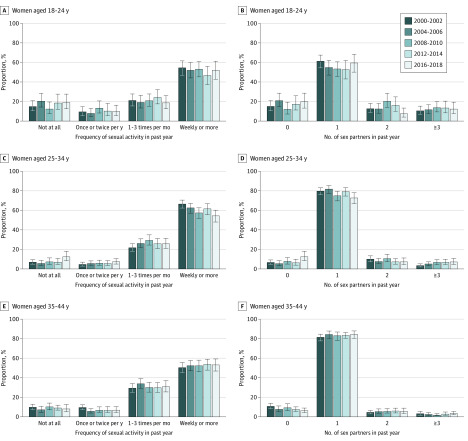
Trends in Frequency of Sexual Activity and Number of Sexual Partners in the Past Year Among US Women by Age Group. Error bars represent 95% CIs.

Among women, the distribution of sexual activity in the total age range remained stable during the study period (Figure 2 and Figure 4 and eFigure 2, eTable 8, and eTable 9 in the Supplement). When analyzed by age group, sexual inactivity increased among women aged 25 to 34 (7.0% vs 12.6%; aOR, 1.17; 95% CI, 1.01-1.35), which coincided with a decrease in weekly or more sexual activity (66.4% vs 54.2%; aOR, 0.90; 95% CI, 0.84-0.96). There was a trend toward an increase in the proportion of individuals reporting 3 or more partners (5.0% vs 7.1%; aOR, 1.10; 95% CI, 1.00-1.20), which was driven by women aged 25 to 34 years (3.5% vs 7.3%; aOR, 1.20; 95% CI, 1.05-1.36) (Figure 4).

Sexual inactivity was rare across all time points and increased only slightly among married men (0.4% vs 1.7%; aOR, 1.36; 95% CI, 1.09-1.69); most of the increase in sexual inactivity occurred among unmarried men (16.2% vs 24.4%; aOR, 1.14; 95% CI, 1.04-1.25) (eFigure 3, eTable 10, and eTable 11 in the Supplement). Decrease in weekly or more sexual activity occurred among both unmarried and married men, with the decrease among married men coinciding with an increase in a sexual frequency of 1 to 3 times per month. Although sexual activity was largely unchanged among unmarried women, a decrease in weekly or more sexual activity and an increase in a sexual frequency of 1 to 3 times per month were observed among married women (eFigure 3, eTable 10, and eTable 11 in the Supplement).

### Factors Associated With Sexual Activity

Associations of sexual inactivity with sociodemographic and behavioral variables are given in the Table; several significant associations were identified. For example, compared with men working full time, those working part time, those who were not working, and students were more likely to be sexually inactive. Men with higher income had a lower likelihood of being sexually inactive. Among women, being a student was associated with sexual inactivity, whereas no significant associations were observed for other categories of employment status or income level. aORs for having no sexual partners were similar to those for sexual inactivity (eTable 12 in the Supplement). The associations for weekly or more sexual activity were largely in the opposite direction of those for sexual inactivity, although higher income was not associated with weekly or more sexual activity among men (eTable 13 in the Supplement). aORs for having 3 or more sexual partners are given in eTable 14 in the Supplement. Black men (vs white men) and men and women identifying as gay, lesbian, or bisexual (vs heterosexual) were more likely to report 3 or more sexual partners. Use of pornographic material was associated with a lower likelihood of sexual inactivity among both men and women.

**Table.** Association Between Sociodemographic and Behavioral Variables and Sexual Inactivity in the General Social Surveys, 2000-2018^a^

Variable	Men (n = 4291)			Women (n = 5213)		
	Total, % (95% CI)	aOR^b^	No. (unweighted/weighted)	Total, % (95% CI)	aOR^b^	No. (unweighted/weighted)
Race						
White	11.6 (10.3-13.1)	1.00 [Reference]	3185/3429	9.8 (8.6-11.1)	1.00 [Reference]	3628/3794
Black	9.1 (6.7-12.3)	0.67 (0.47-0.97)	573/611.2	11.9 (9.5-14.7)	1.21 (0.91-1.61)	989/876.9
Other	14.3 (10.9-18.5)	1.13 (0.81-1.58)	533/635	12.0 (9.0-15.7)	1.20 (0.85-1.70)	596/670.6
Sexual orientation^c^						
Heterosexual	12.9 (11.2-14.9)	1.00 [Reference]	2079/2305	10.7 (9.3-12.4)	1.00 [Reference]	2419/2520
Gay, lesbian, or bisexual	18.6 (11.5-28.6)	1.40 (0.76-2.57)	106/98.1	10.5 (6.5-16.6)	0.86 (0.50-1.49)	206/196.2
Religion						
None	12.9 (10.7-15.5)	1.00 [Reference]	1178/1257	9.5 (7.6-11.7)	1.00 [Reference]	1037/1053
Christian	10.3 (9.1-11.8)	0.89 (0.69-1.15)	2841/3115	10.2 (9.1-11.4)	1.21 (0.93-1.56)	3877/3971
Non-Christian	20.5 (15.0-27.5)	1.92 (1.23-3.01)	257/284.5	16.1 (11.5-22.2)	1.93 (1.20-3.12)	287/307.1
Educational level						
High school or less	12.5 (11.0-14.2)	1.00 [Reference]	2773/3122	11.0 (9.8-12.4)	1.00 [Reference]	3325/3478
College or more	9.8 (8.2-11.7)	0.99 (0.77-1.26)	1515/1549	9.2 (7.7-10.8)	0.91 (0.72-1.14)	1881/1856
Employment						
Full time	7.7 (6.6-8.9)	1.00 [Reference]	3082/3223	9.3 (8.1-10.7)	1.00 [Reference]	2620/2589
Part time	19.2 (15.1-24.1)	2.08 (1.48-2.93)	400/498.7	10.3 (8.0-13.2)	1.05 (0.77-1.44)	831/883.5
Student	28.2 (22.9-34.2)	2.94 (2.06-4.21)	296/379	23.3 (18.6-28.9)	2.37 (1.68-3.35)	381/426.7
Not working	15.8 (12.2-20.2)	2.08 (1.48-2.91)	444/509.4	7.3 (5.8-9.2)	0.77 (0.58-1.04)	1306/1359
Annual income, US$^d^						
0-9999	27.6 (20.7-35.7)	1.00 [Reference]	210/259.8	12.6 (9.2-17.1)	1.00 [Reference]	372/434.2
10 000-49 000	11.5 (7.4-17.3)	0.44 (0.23-0.84)	253/262.1	14.2 (9.5-20.8)	1.38 (0.77-2.45)	277/275.3
≥50 000	7.1 (3.7-13.4)	0.37 (0.15-0.90)	179/187.8	5.6 (2.5-11.9)	0.63 (0.26-1.54)	102/101.9
Steady partner^e^						
No	31.7 (26.2-37.7)	1.00 [Reference]	339/382.4	33.7 (27.7-40.4)	1.00 [Reference]	316/311.5
Yes	3.6 (1.9-6.7)	0.10 (0.05-0.21)	346/379.6	1.4 (0.7-2.9)	0.02 (0.01-0.06)	669/734.8
Marital status						
Married	1.9 (1.3-2.8)	1.00 [Reference]	1891/1674	1.1 (0.7-1.7)	1.00 [Reference]	2504/2183
Previously married	10.0 (7.4-13.5)	5.94 (3.51-10.05)	432.8/510	13.2 (10.9-16.0)	13.29 (8.02-22.02)	709.8/905
Never married	19.8 (17.8-22.0)	11.65 (7.56-17.96)	2351/2105	20.4 (18.3-22.7)	28.00 (17.45-44.92)	2127/2125
Region						
Northeast	13.0 (10.5-16.0)	1.00 [Reference]	676/768.4	12.0 (9.5-14.9)	1.00 [Reference]	866/924.3
Midwest	11.7 (9.3-14.7)	0.88 (0.61-1.25)	1072/1117	8.6 (6.7-10.9)	0.67 (0.46-0.97)	1218/1198
South	10.0 (8.3-12.1)	0.76 (0.55-1.04)	1497/1621	9.7 (8.2-11.5)	0.77 (0.56-1.05)	1919/1910
West	13.0 (10.7-15.6)	0.95 (0.68-1.32)	1046/1169	11.9 (9.9-14.4)	0.95 (0.68-1.33)	1210/1309
Residence (population size)						
City (>250 000)	12.1 (9.7-14.9)	1.00 [Reference]	875/903.4	14.7 (12.1-17.7)	1.00 [Reference]	1033/1034
City (50-250 000)	11.9 (9.4-14.9)	1.01 (0.72-1.43)	891/939.1	11.0 (8.9-13.4)	0.73 (0.53-1.00)	1124/1121
Suburbs or small city	12.0 (10.4-13.9)	1.05 (0.79-1.40)	1904/2169	9.9 (8.5-11.6)	0.65 (0.49-0.86)	2256/2377
Rural	9.5 (7.1-12.6)	0.86 (0.58-1.29)	621/663.6	5.6 (4.0-7.7)	0.35 (0.23-0.53)	800/808.9
Pornography use in last year						
No	15.3 (13.0-18.0)	1.00 [Reference]	1390/1273	13.5 (11.9-15.2)	1.00 [Reference]	2478/2371
Yes	10.4 (8.6-12.4)	0.5 (0.38-0.66)	1543/1425	4.6 (3.1-6.7)	0.28 (0.18-0.43)	899.4/917
Internet use per week, h^f^						
0 to <5	9.5 (7.5-12.0)	1.00 [Reference]	1057/960	9.6 (7.9-11.7)	1.00 [Reference]	1448/1389
≥5 to <15	9.9 (7.5-12.8)	0.93 (0.62-1.38)	930.2/849	12.8 (10.4-15.7)	1.34 (0.97-1.86)	944.7/920
≥15	15.5 (12.3-19.2)	1.44 (0.97-2.13)	709.3/648	12.0 (9.3-15.4)	1.25 (0.84-1.85)	664.7/642
Time worked per week, h						
0	21.3 (18.0-25.0)	1.00 [Reference]	879.6/730	11.1 (9.4-13.1)	1.00 [Reference]	1755/1661
1-20	20.3 (14.5-27.9)	0.94 (0.59-1.51)	231.2/187	11.0 (7.8-15.5)	0.96 (0.62-1.49)	419.8/390
21-40	10.1 (8.4-12.1)	0.50 (0.37-0.67)	1613/1481	9.5 (8.0-11.2)	0.88 (0.68-1.14)	2063/2040
41-59	6.7 (5.3-8.5)	0.37 (0.26-0.52)	1249/1207	8.8 (6.8-11.3)	0.83 (0.59-1.18)	765.1/777
≥60	6.3 (4.4-8.9)	0.34 (0.22-0.54)	614.8/596	11.7 (7.5-17.7)	1.22 (0.73-2.04)	225.6/237

Abbreviation: aOR, age-adjusted odds ratio.

^a^ Analyses included all participants who had been asked the question regarding the variable of interest. Missing variables were excluded by variable. For men, missing values were religion (n = 15), sexual orientation (n = 15), annual income (n = 8), educational level (n = 3), employment (n = 69), stable relation (n = 3), marital status (n = 2), pornography use (n = 12), internet use per week (n = 35), and time worked per week (n = 90). For women, missing values were religion (n = 12), sexual orientation (n = 25), educational level (n = 7), annual income (n = 10), stable relation (n = 1), employment (n = 75), pornography use (n = 12), internet use per week (n = 64), and time worked per week (n = 108).

^b^ aORs were calculated using a logistic regression model with sexual inactivity as the binary outcome variable (yes = 1, no = 0) and age (continuous) and survey period (categorical) as independent variables.

^c^ Using General Social Surveys of 2008 to 2018.

^d^ Using General Social Surveys of 2016 to 2018.

^e^ Using General Social Surveys of 2012 to 2018.

^f^ Not including the General Social Survey of 2008.

### Additional Analyses and Post Hoc Analyses

Findings regarding trends in sexual frequency and number of sexual partners were similar when analyzing each survey year separately (eFigure 4 and eFigure 5 in the Supplement). In the post hoc analyses, an increase in sexual inactivity and having no sexual partners among men was observed in most sociodemographic subgroups but not among gay or bisexual participants (sexual inactivity: aOR for trend, 0.62; 95% CI, 0.29-1.33; *P* = 0.05 for interaction vs heterosexual participants; no sexual partner: aOR, 0.52; 95% CI, 0.26-1.03; *P* = 0.009 for interaction) (eTable 15 and eTable 16 in the Supplement). Similarly, the decline in weekly or more sexual activity among men was observed across subgroups, although trends did not differ by sexual orientation (eTable 17 in the Supplement). In multivariable logistic regression models adjusted for sociodemographic variables, including changes in the proportion married, the aORs for the trend remained largely similar to those in the primary analyses for sexual inactivity, no sexual partners, and weekly or more sexual activity among men (eTable 18 in the Supplement). The distribution of sexual frequency and number of sexual partners among participants identifying as gay, lesbian, or bisexual is shown in eFigure 6 in the Supplement.

## Discussion

Using US nationally representative survey data, we estimated that 30.9% of men and 19.1% of women aged 18 to 24 years in 2016-2018 reported being sexually inactive in the past year, with these proportions being similar in both sexes among those aged 25 to 34 years (14.1% vs 12.6%) and 35 to 44 years (8.0% vs 8.5%). Between 2000 and 2018, sexual inactivity increased among men aged 18 to 24 years and among men and women aged 25 to 34 years. The increase in sexual inactivity coincided with decreases in the proportion reporting sexual activity at least weekly or 1 sexual partner and occurred mainly among unmarried men. Among married men and women, there was a decrease in sexual activity at least weekly, whereas sexual inactivity was rare and did not change substantially. Men with lower income and with part-time or no employment were more likely to be sexually inactive, as were men and women who were students.

Few studies have investigated recent trends in sexual inactivity in national populations. An earlier analysis^15^ of 20- to 24-year-old women and men in the General Social Survey from 1989 to 2014 found that the proportion reporting sexual inactivity was larger for those born in 1990 to 1994 (15.2%) than for those born in 1980 to 1989 (11.7%) and 1970 to 1979 (11.5%). Moreover, in an analysis^15^ of the full age range (18-96 years) that controlled for age and period, the proportion reporting no sexual partners after 18 years of age was larger among those born in the 1990s than among those born in the 1960s, 1970s, and 1980s, although data for those born in the 1990s were only available up to the age of 24 years. These findings align with trends observed in our sex-specific analyses in more recent surveys, which assessed a broader range of age groups and measures of sexual activity. In an analysis of nationally representative data from Germany, the proportion of men 18 years or older who reported no sexual activity in the past year increased between 2005 and 2016.^20^ Consistent with our findings, the increase in Germany mainly occurred among men living without a partner and among men aged 18 to 30 years; in this age group, sexual inactivity increased from 7.5% to 20.3%. In contrast, in national surveys of 16- to 44-year-old adults in Britain, the proportion of men and women reporting no sexual activity in the past year remained stable between 2000 and 2011.^18^

Consistent with our findings of a decrease in sexual activity at least weekly among married men and women living in the US, decreases in the mean sexual frequency among married or partnered individuals have been demonstrated in previous analyses of the General Social Survey (1989-2014, individuals aged ≥18 years)^16^ and in national surveys in Finland (1999 vs 2007, individuals aged 18-54 years),^21^ Australia (2001-2002 vs 2012-2013, individuals aged 16-59 years),^22^ and Britain (2000 vs 2011, individuals aged 16-44 years).^12^ Although our analyses found an increase in sexual inactivity among unmarried men, these previous studies^12,16,21,22^ did not find decreases in the mean sexual frequency among unpartnered individuals. Although this apparent discrepancy can be explained by our use of more recent data and differences in the studied populations and age and sex groups, it is also possible that changes in the distribution of sexual frequency might have gone unnoticed in analyses of mean sexual frequency. Of importance, although the mean sexual frequency among those who were sexually active may reflect their priorities and preferences, sexual inactivity may reflect an absence of sexually intimate relationships, with substantially different implications for public health and society. As such, our study highlights the importance of assessing the distribution of sexual activity in populations rather than just the mean frequency, especially given the increasing number of unpartnered individuals.

Several hypotheses for why individuals engage in less sexual activity have been proposed. Although theories regarding the use of pornography and longer working hours were not supported by our analyses, plausible reasons include changes in sexual norms that may affect actual and reported sexual activity; the stress and busyness of modern life in which leisure, work, and intimate relationships need to be juggled^12,23^; and the supply of online entertainment that may compete with sexual activity.^12,16,23,24^ Although these hypotheses could explain the decrease in frequency among partnered individuals, additional mechanisms may be associated with the increases in sexual inactivity observed in our study. For example, rates of depression and anxiety have increased among young US adults; US adolescents are increasingly postponing the start of adult activities, including sex and dating^25^; and it has been hypothesized that the introduction of smartphones has resulted in less opportunity for and skills in real-world human interactions.^26^ For women, sexual inactivity may also be associated with a greater prevalence of “hooking up” (which has generally been reported to be less pleasurable for women)^27^ or potential increases in sexual aggression directed toward women.^28^ Moreover, we found that men with lower income and those with part-time or no employment were more likely to be sexually inactive. These findings are consistent with literature showing associations between lower income and measures of sexual inactivity^12,13,14,29^ and decreased appeal in the mating market for men.^30,31,32,33^ Given the widening disparities in economic security (some of which are more pronounced among young men),^34^ the preference for men of higher socioeconomic status, and the larger number of college-educated young women than men in the US, it has been suggested that a subset of young men find it difficult to establish themselves in the heterosexual mating market.^35,36,37^ In our study, sexual inactivity in younger age groups was more common among men than women, and the increase in sexual inactivity was observed only among men identifying as heterosexual, although educational level was not associated with any measure of sexual activity, and being a student was associated with sexual inactivity among men and women. Moreover, the increase in sexual inactivity among men remained after adjustment for changes in employment status.

### Limitations

This study has limitations. First, we used survey data, which are subject to response and reporting bias. Second, because the data were cross-sectional, we could not assess temporality of the associations between sociodemographic factors and measures of sexual activity. Third, sexual activity was not defined in the General Social Survey. Thus, some participants may have interpreted the terms *have sex* and *sex partners* using a definition of vaginal intercourse (or *sex partners* as referring only to relational partners), whereas others may have considered *sex* to include oral sex or mutual masturbation.^38,39,40^ Some studies^38,41^ have found that men are more likely than women to report nonpenetrative sex as *sex*. As such, differences between the sexes and potential changes over time in the interpretation of the survey questions may have affected our findings. Fourth, we could not assess reasons for sexual inactivity and to what extent this was associated with satisfaction or dissatisfaction. In a US study,^29^ sexually inactive individuals reported similar happiness levels as did those who were sexually active. In a British national survey, less than half of the sexually inactive participants aged 16 to 74 years reported dissatisfaction with their sex life.^14^ Some individuals report never having felt sexual attraction to anyone^42^ or a lack of interest in sex,^43^ whereas others have difficulties in finding sexual partners, with this being a cause of distress.^44^ To place our findings into context, further studies are needed on reasons for and potential feelings about sexual inactivity.

## Conclusions

 This survey study found that from 2000 to 2018 sexual inactivity increased among US men such that approximately 1 in 3 men aged 18 to 24 years reported no sexual activity in the past year. Sexual inactivity also increased among men and women aged 25 to 34 years, with the increase among men mainly occurring among unmarried individuals.

## Notes

Supplement. **eAppendix.** The General Social Survey **eTable 1.** Tabulation of Participants in the 2012 Dataset by Their Coded Response of the Questions Regarding Sexual Frequency and Number of Sexual Partners in the Last Year **eTable 2.** Number of Survey Participants Who Were Excluded and Included in the Analyses in Each Survey Year **eTable 3.** Definitions and Categorization of Sociodemographic Variables **eTable 4.** Weighted Characteristics of the Study Populations from the General Social Survey, 2000-2018, for the Analyses of the Sexual Frequency and Number of Sexual Partners in the Last Year **eTable 5.** Sexual Frequency and Number of Sexual Partners in the Last Year Among Men and Women in 2016/2018 **eTable 6.** Trends in Sexual Frequency in the Last Year Among Men in the Total Age Range and by Age Group **eTable 7.** Trends in the Number of Sexual Partners in the Last Year Among Men in the Total Age Range and by Age Group **eTable 8.** Trends in Sexual Frequency in Last Year Among Women in the Total Age Range and by Age Group **eTable 9.** Trends in the Number of Sexual Partners in the Last Year Among Women in the Total Age Range and by Age Group **eTable 10.** Trends in Sexual Frequency Among Men and Women Aged 18-44 Years by Marital Status **eTable 11.** Trends in Number of Sexual Partners in Last Year Among Men and Women Aged 18-44 Years by Marital Status **eTable 12.** Association Between Sociodemographic and Behavioral Variables and No Sexual Partners in the Last Year **eTable 13.** Association Between Sociodemographic and Behavioral Variables and ≥Weekly Sex in the Last Year **eTable 14.** Association Between Sociodemographic and Behavioral Variables and Having 3 or More Sexual Partners in the Last Year **eTable 15.** Trends in Sexual Inactivity in Last Year Among Men in Sociodemographic Subgroups **eTable 16.** Trends in Having No Sexual Partner in the Last Year Among Men in Sociodemographic Subgroups **eTable 17.** Trends in Weekly Sex in Last Year Among Men in Sociodemographic Subgroups **eTable 18.** Odds ratios of Survey Period for Sexual Inactivity, Weekly Sex, and No Sexual Partners in the Last Year Among Men in Logistic Regression Models, With and Without Adjustment for Sociodemographic Variables **eFigure 1.** Distribution of Frequency of Sex and Number of Sexual Partners in the Last Year Among US Men and Women Aged 18-44 in the General Social Survey, 2016-2018 **eFigure 2.** The Proportion of Men and Women Who Were Sexually Inactive in the Last Year by Survey Period and Age **eFigure 3.** Trends in Sexual Frequency and Number of Sexual Partners in the Last Year Among US Men and Women, Aged 18-44 Years, by Marital Status **eFigure 4.** Frequency of Sex in Last Year by Survey Year **eFigure 5.** Number of Sexual Partners in Last Year by Survey Year **eFigure 6.** Frequency of Sex and the Number of Sexual Partners in the Last Year Among Participants Identifying as Gay, Lesbian, or Bisexual **eReferences**
