# Supplementary material for: Trends in Frequency of Sexual Activity and Number of Sexual Partners Among Adults Aged 18 to 44 Years in the US, 2000-2018
Source: JAMA Netw Open. 2020 Jun 12;3(6):e203833. doi: 10.1001/jamanetworkopen.2020.3833 (PMC7293001; doi:10.1001/jamanetworkopen.2020.3833)
Supplement: Supplement. — eAppendix. The General Social Survey eTable 1. Tabulation of Participants in the 2012 Dataset by Their Coded Response of the Questions Regarding Sexual Frequency and Number of Sexual Partners in the Last Year eTable 2. Number of Survey Participants Who Were Excluded and Included in the Analyses in Each Survey Year eTable 3. Definitions and Categorization of Sociodemographic Variables eTable 4. Weighted Characteristics of the Study Populations from the General Social Survey, 2000-2018, for the Analyses of the Sexual Frequency and Number of Sexual Partners in the Last Year eTable 5. Sexual Frequency and Number of Sexual Partners in the Last Year Among Men and Women in 2016/2018 eTable 6. Trends in Sexual Frequency in the Last Year Among Men in the Total Age Range and by Age Group eTable 7. Trends in the Number of Sexual Partners in the Last Year Among Men in the Total Age Range and by Age Group eTable 8. Trends in Sexual Frequency in Last Year Among Women in the Total Age Range and by Age Group eTable 9. Trends in the Number of Sexual Partners in the Last Year Among Women in the Total Age Range and by Age Group eTable 10. Trends in Sexual Frequency Among Men and Women Aged 18-44 Years by Marital Status eTable 11. Trends in Number of Sexual Partners in Last Year Among Men and Women Aged 18-44 Years by Marital Status eTable 12. Association Between Sociodemographic and Behavioral Variables and No Sexual Partners in the Last Year eTable 13. Association Between Sociodemographic and Behavioral Variables and ≥Weekly Sex in the Last Year eTable 14. Association Between Sociodemographic and Behavioral Variables and Having 3 or More Sexual Partners in the Last Year eTable 15. Trends in Sexual Inactivity in Last Year Among Men in Sociodemographic Subgroups eTable 16. Trends in Having No Sexual Partner in the Last Year Among Men in Sociodemographic Subgroups eTable 17. Trends in Weekly Sex in Last Year Among Men in Sociodemographic Subgroups eTable 18. Odds ratios of Survey Period [file jamanetwopen-3-e203833-s001.pdf]

Supplementary Online Content

Ueda P, Mercer CH, Ghaznavi C, Herbenick D. Trends in frequency of sexual activity and number of sexual partners among adults aged 18 to 44 years in the US, 2000-2018. *JAMA Netw Open*. 2020;3(6):e203833. doi:10.1001/jamanetworkopen.2020.3833

eAppendix. The General Social Survey
eTable 1. Tabulation of Participants in the 2012 Dataset by Their Coded Response of the Questions Regarding Sexual Frequency and Number of Sexual Partners in the Last Year
eTable 2. Number of Survey Participants Who Were Excluded and Included in the Analyses in Each Survey Year
eTable 3. Definitions and Categorization of Sociodemographic Variables
eTable 4. Weighted Characteristics of the Study Populations from the General Social Survey, 2000-2018, for the Analyses of the Sexual Frequency and Number of Sexual Partners in the Last Year
eTable 5. Sexual Frequency and Number of Sexual Partners in the Last Year Among Men and Women in 2016/2018
eTable 6. Trends in Sexual Frequency in the Last Year Among Men in the Total Age Range and by Age Group
eTable 7. Trends in the Number of Sexual Partners in the Last Year Among Men in the Total Age Range and by Age Group
eTable 8. Trends in Sexual Frequency in Last Year Among Women in the Total Age Range and by Age Group
eTable 9. Trends in the Number of Sexual Partners in the Last Year Among Women in the Total Age Range and by Age Group
eTable 10. Trends in Sexual Frequency Among Men and Women Aged 18-44 Years by Marital Status
eTable 11. Trends in Number of Sexual Partners in Last Year Among Men and Women Aged 18-44 Years by Marital Status
eTable 12. Association Between Sociodemographic and Behavioral Variables and No Sexual Partners in the Last Year
eTable 13. Association Between Sociodemographic and Behavioral Variables and ≥Weekly Sex in the Last Year
eTable 14. Association Between Sociodemographic and Behavioral Variables and Having 3 or More Sexual Partners in the Last Year
eTable 15. Trends in Sexual Inactivity in Last Year Among Men in Sociodemographic Subgroups
eTable 16. Trends in Having No Sexual Partner in the Last Year Among Men in Sociodemographic Subgroups
eTable 17. Trends in Weekly Sex in Last Year Among Men in Sociodemographic Subgroups
eTable 18. Odds ratios of Survey Period for Sexual Inactivity, Weekly Sex, and No Sexual Partners in the Last Year Among Men in Logistic Regression Models, With and Without Adjustment for Sociodemographic Variables
eFigure 1. Distribution of Frequency of Sex and Number of Sexual Partners in the Last Year Among US Men and Women Aged 18-44 in the General Social Survey, 2016-2018
eFigure 2. The Proportion of Men and Women Who Were Sexually Inactive in the Last Year by Survey Period and Age
eFigure 3. Trends in Sexual Frequency and Number of Sexual Partners in the Last Year Among US Men and Women, Aged 18-44 Years, by Marital Status
eFigure 4. Frequency of Sex in Last Year by Survey Year
eFigure 5. Number of Sexual Partners in Last Year by Survey Year
eFigure 6. Frequency of Sex and the Number of Sexual Partners in the Last Year Among Participants Identifying as Gay, Lesbian, or Bisexual
eReferences

This supplementary material has been provided by the authors to give readers additional information about their work.

© 2020 Ueda P et al. *JAMA Network Open.*

eAppendix. The General Social Survey

The General Social Survey (GSS) is a nationally representative survey which has assessed attitudes and behaviors in the US population since 1972. GSS includes a standard core of demographic, behavioral and attitudinal questions, as well as topics of special interest such as stress, psychological well-being and crime and violence.[1, 2] The survey is conducted by NORC at the University of Chicago, which participates in the American Association for Public Opinion Research (AAPOR)'s Transparency Initiative, and follows best practices in survey research.[3] Response rates for the surveys included in our study, which were calculated according to the recommendations of the AAPOR, [4] were: 70.0% (2000), 70.1% (2002), 70.4% (2004), 71.2% (2006), 70.4% (2008), 70.3% (2010), 71.4% (2012), 60.2% (2014), 61.3% (2016) and 59.5% (2018).[2] The GSS data are provided online by NORC at the University of Chicago (gss.norc.org). Additional data from the analyses performed in this study are available upon request from the corresponding author.

© 2020 Ueda P et al. *JAMA Network Open.*

Survey questions used to categorize sexual frequency and number of sexual partners in the last year

About how often did you have sex during the last 12 months?

- Not at all
- Once or twice
- Once a month
- 2-3 times a month
- Weekly
- 2-3 per week
- ≥4 per week
- Don’t know

How many sex partners have you had in the last 12 months?

- No partners
- 1 partner
- 2 partners
- 3 partners
- 4 partners
- 5-10 partners
- 11-20 partners
- 21-100 partners
- More than 100 partners
- 1 or more, # unknown
- Don’t know

© 2020 Ueda P et al. *JAMA Network Open.*

Categorization of sexual frequency and the number of sexual partners

The categories "sexually inactive" and "no sexual partners" were used in order to address one of the aims of the study, which included assessing the proportion of adults being sexually inactive or having no sexual partner. Those having sex weekly or more were considered as having sex at a regular frequency, with this categorization also being used by a previous analysis of the British Natsal study.[5] A sexual frequency of 1-3 times/month was considered as a separate category from 1-2 times/year as the former may represent a more stable sexual frequency than the latter, which may indicate more sporadic and infrequent sexual encounters, although this may not hold true for all individuals. The use of the category of sex more than once a month has also been used in previous analyses of the Natsal study.[6] The categorization of those with one or more sexual partners was based on a preliminary inspection of the data showing that few individuals (around 6%) had 4 or more partners, and further categorization of those with several sexual partners would not allow for meaningful statistical analysis given the sample size.

Categorization of sexual frequency in the 2012 dataset

All survey participants who were asked the question regarding the sexual frequency in the last year were also asked about the number of sexual partners they had in the last year. We noticed a probable coding error in the 2012 dataset such that all participants who had responded that they had no sexual partners in the last year had been coded as if they had not been asked the question about sexual frequency. eTable 1 shows the tabulation of participants by their coded responses to the question regarding sexual frequency and number of sexual partners. We considered the n=102 participants who

© 2020 Ueda P et al. *JAMA Network Open.*

had answered “no partners” on the question regarding number of sexual partners but who had been coded as not having been asked to answer the question regarding sexual frequency, as having answered “not at all” to this question.

© 2020 Ueda P et al. *JAMA Network Open.*

eTable 1. Tabulation of Participants in the 2012 Dataset by Their Coded Response of the Questions Regarding Sexual Frequency and Number of Sexual Partners in the Last Year

	Sexual frequency										
Number of partners	"not at all"	Once or twice	Once a month	2-3 times a month	Weekly	2-3 times per week	≥4 times per week	Don't know	Inapplicable[a]	Not answered	Total
no partners	0	0	0	0	0	0	0	0	102	0	102
1 partner	6	30	48	98	90	177	58	10	0	25	542
2 partners	0	13	8	11	9	18	18	0	0	1	78
3 partners	2	4	3	11	5	7	3	0	0	0	35
4 partners	0	0	4	6	10	5	3	0	0	0	28
5-10 partners	0	0	2	3	5	8	5	0	0	0	23
11-20 partners	0	1	0	1	0	1	0	0	0	1	4
21-100 partners	0	0	0	0	1	1	2	0	0	0	4
1 or more, # unknown	0	0	0	0	0	0	0	1	0	6	7
Inapplicable[a]	0	0	0	0	0	0	0	0	64	0	64
Not answered	0	0	0	0	0	0	0	0	0	4	4
Total	8	48	65	130	120	217	89	11	166	37	891

[a] Respondents who are not asked to answer a specific question are assigned to IAP (Inapplicable).

© 2020 Ueda P et al. *JAMA Network Open.*

eTable 2. Number of Survey Participants Who Were Excluded and Included in the Analyses in Each Survey Year

	Sexual frequency[a]		*Number of sexual partners*	
	Excluded	*Included*	*Excluded*	*Included*
2000	48	1,256	19	1,280
2002	50	1,154	20	1,183
2004	53	1,125	20	1,156
2006	38	1,119	14	1,142
2008	40	796	12	823
2010	29	832	12	849
2012	48	779	11	816
2014	48	1,007	22	1,033
2016	22	793	7	809
2018	19	643	4	658
Total	395	9,504	141	9,749

[a] n=14 participants with unknown sexual frequency who had answered that they had no sexual partners in the last year were categorized as sexually inactive.

[b] n=5 participants with unknown number of sexual partners who had answered that they were sexually inactive in the last year were categorized as having no sexual partners..

Survey participants were excluded if they had been asked the question regarding sexual frequency and number of sexual partners, respectively, and had missing data such that they could not be categorized according to the categories of sexual activity/number of sexual partners used in the analyses.

© 2020 Ueda P et al. *JAMA Network Open.*

eTable 3. Definitions and Categorization of Sociodemographic Variables

Variable	Definition/categorization	References [a]
Education (completed)	1. High school of less<br>2. Junior college or more	[7–13]
Annual income in USD (2016-2018) [b]	1. 0-9,999<br>2. 10,000-49,999<br>3. 50,000-74,999<br>4. ≥75,000	[7–13]
Employment status	1. Working full-time<br>2. Working part-time<br>3. Student<br>4. Not working (including: "temporary not working", "unemployed, laid off", "retired", "keeping house")	[7–13]
Race	1. White<br>2. Black<br>3. Other	[11, 14, 15]
Region	1. Northeast<br>2. Midwest<br>3. South<br>4. West	[12, 16]
Area of residence (categorization based on[17])	1. Rural areas and small towns (town with population more than 2,500 and less than 10,000 or in smaller areas or open country)<br>2. Suburbs or small towns (suburbs of medium or large cities, or in towns with population between 10,000 and 50,000)<br>3. Medium cities (population of 50,000 to <250,000)<br>4. Large cities (population ≥250,000)	[12, 13, 16]
Marital status	1. Married (married according to "marital" variable, or "living as married according to "posslq" (2012-2018) variable.<br>2. Previously married (widowed; divorced, according to the "marital" variable AND not married as defined above)<br>3. Never married, according to the "marital" variable AND not married as defined above)<br><br>For the subgroup analyses by marital status, categories (2) and (3) were considered as one group.	[5, 18, 19]
Relationship status (2012-2018)	1. No stable partner<br>2. Stable partner (Married; stable partner, not living together; stable partner, living together)	[5, 18, 19]
Sexual orientation (2008-2018)	1. "Heterosexual or straight"<br>2. "Gay, lesbian, or homosexual" or "bisexual"	[20]
Religion	1. None<br>2. Christian<br>3. Other religion.	[21]
Pornography use	1. No<br>2. Yes<br><br>Categorized based on the question: "Have you seen an X-rated movie in the past year?"	[18]
Hours of internet use per week (2000-2006; 2010-2018)	1. 0-5<br>2. ≥5 to <15<br>3. ≥15	[5, 18, 22, 23]

© 2020 Ueda P et al. *JAMA Network Open.*

Number of hours worked per week	1. 0<br>2. 1 to <20<br>3. 21 to 40<br>4. 41 to 59<br>5. 60 or more	[5, 18]

[a] References in which association with measures of sexual activity or related factors have been shown or suggested.

[b] All participants were asked the question regarding annual income if they had responded that they had an income from an occupation. Participants not answering the question regarding annual income as they responded that they did not have an income were categorized as having an income of 0 USD. The question providing detailed data on the distribution of incomes of 25000 USD or more was only available for the years 2016 and 2018.

© 2020 Ueda P et al. *JAMA Network Open.*

eTable 4. Weighted Characteristics of the Study Populations from the General Social Survey, 2000-2018, for the Analyses of the Sexual Frequency and Number of Sexual Partners in the Last Year

Numbers are shown in percent.

	Sexual frequency		Number of sexual partners	
**Age group**	**Men**	**Women**	**Men**	**Women**
18-24	25.4	22.5	25.1	22.0
25-34	38.6	38.1	38.4	38.1
35-44	36.0	39.5	36.5	39.9
**Race**				
White	73.3	71.0	73.5	71.1
Black	13.1	16.4	12.9	16.3
Other	13.6	12.6	13.6	12.6
**Sexual orientation**[a]				
Heterosexual	95.9	92.8	95.9	93
Gay/lesbian/bisexual	4.1	7.2	4.1	7.0
**Religion**				
None	27.0	19.7	26.8	19.6
Christian	66.9	74.5	66.9	74.6
Non-Christian	6.1	5.8	6.3	5.8
**Education**				
High school or less	66.8	65.2	66.5	64.9
College or more	33.2	34.8	33.5	35.1
**Employment**				
Fulltime	69.9	49.2	70.2	49.1
Part-time	10.8	16.8	10.7	16.9
Student	8.2	8.1	8.1	7.9
Not working	11.0	25.8	11.0	26.1
**Annual income (USD)**[b]				
0-9,999	36.6	53.5	36.4	54.1
10,000-49,999	36.9	33.9	36.6	33.8
≥50,000	26.5	12.6	26.9	12.1
**Steady partner**[c]				
No	50.2	29.8	49.1	29.1
Yes	49.8	70.2	50.9	70.9
**Marital status**				
Married	40.4	46.9	41.1	47.6
Previously married	9.3	13.3	9.1	13.2
Never married	50.3	39.8	49.7	39.2
**Region**				
Northeast	16.4	17.3	16.5	17.7
Midwest	23.9	22.4	23.8	22.3
South	34.7	35.8	34.6	35.7
West	25.0	24.5	25.1	24.3
**Residence (population size)**				
City >250,000.	19.3	19.4	19.4	19.4

© 2020 Ueda P et al. *JAMA Network Open.*

City 50-250,000	20.1	21.0	20.2	21.1
Suburbs/small city	46.4	44.5	46.3	44.4
Rural	14.2	15.1	14.1	15.1
**Porn use in last year**				
No	47.4	73.4	47.9	74
Yes	52.6	26.6	52.1	26
**Hours of internet use per week**[d]				
0-5	39.2	47.4	39.2	47.6
≥5 to <15	34.5	30.9	34.4	30.9
≥15	26.3	21.7	26.4	21.5
**Hours worked per week**				
0	19.2	33.6	19	33.7
1-20	5	8	5	8.1
21-40	35.2	39.5	35.2	39.6
41-60	27.2	14.6	27.1	14.2
≥60	13.4	4.3	13.7	4.4
N weighted/unweighted	4291/4676	5213/5341	4372/4766	5377/5527

Analyses included all participants who had been asked the question regarding the variable of interest. Missing values and number of observations for each variable are shown in eTables 12 and 13.

a. Using General Social Surveys of 2008-2018.
b. Using General Social Surveys of 2016-2018.
c. Using General Social Surveys of 2012-2018.
d. Not including the General Social Survey of 2008.

© 2020 Ueda P et al. *JAMA Network Open.*

eTable 5. Sexual Frequency and Number of Sexual Partners in the Last Year Among Men and Women in 2016/2018

Weighted estimates used in Figure 1 and eFigure 1.

	**Sexual frequency**				**Number of sexual partners**			
Age group		Men	Women	p		Men	Women	p
**18-44 y**	Not at all	16.5 (13 to 20.7)	12.6 (9.9 to 15.7)	0.0859	0	16.4 (12.9 to 20.6)	12.0 (9.6 to 14.8)	0.0367
	1-2 times/year	9.1 (6.8 to 12.1)	7.9 (6.0 to 10.4)	0.504	1	57.5 (52.3 to 62.5)	74.2 (70.6 to 77.5)	<0.001
	1-3 times/month	27.7 (24.3 to 31.3)	26.2 (23.0 to 29.6)	0.5463	2	11.6 (9.2 to 14.7)	6.8 (5.0 to 9.1)	0.0027
	Weekly or more	46.7 (42.3 to 51.1)	53.3 (49.3 to 57.3)	0.0197	≥3	14.5 (11.3 to 18.3)	7.1 (5.3 to 9.5)	0.0001
**18-24 y**	Not at all	30.9 (22.7 to 40.5)	19.1 (12.7 to 27.8)	0.0509	0	32.1 (23.7 to 41.7)	20.1 (13.6 to 28.6)	0.032
	1-2 times/year	8.9 (4.8 to 16)	10.1 (6.0 to 16.6)	0.7664	1	30.0 (22.9 to 38.1)	59.7 (50.3 to 68.4)	<0.001
	1-3 times/month	22.7 (16.3 to 30.7)	18.6 (12.8 to 26.2)	0.4106	2	14.7 (9.5 to 22.2)	7.7 (4.3 to 13.5)	0.0541
	Weekly or more	37.4 (28.6 to 47.2)	52.1 (42.9 to 61.2)	0.0196	≥3	23.2 (16.4 to 31.8)	12.5 (7.8 to 19.6)	0.0362
**25-34 y**	Not at all	14.1 (9.7 to 20.0)	12.6 (8.5 to 18.2)	0.6582	0	13.6 (9.2 to 19.7)	12.8 (8.7 to 18.3)	0.8118
	1-2 times/year	7.5 (4.8 to 11.5)	7.4 (5.1 to 10.6)	0.9556	1	58.0 (50.7 to 65.0)	72.7 (66.9 to 77.9)	0.0005
	1-3 times/month	28.1 (22.6 to 34.4)	25.9 (21.1 to 31.3)	0.5829	2	11.6 (7.4 to 17.5)	7.3 (4.6 to 11.2)	0.1168
	Weekly or more	50.3 (44.1 to 56.5)	54.2 (48.2 to 60)	0.3557	≥3	16.8 (11.9 to 23.3)	7.3 (4.8 to 10.9)	0.0004
**35-44 y**	Not at all	8 (5.0 to 12.7)	8.5 (5.5 to 12.9)	0.8765	0	7.8 (4.9 to 12.3)	6.3 (4.4 to 9)	0.4866
	1-2 times/year	11.1 (6.8 to 17.6)	7.1 (4.7 to 10.6)	0.1713	1	77.3 (71.1 to 82.4)	84.3 (80 to 87.8)	0.0372
	1-3 times/month	31.0 (25.1 to 37.6)	31.2 (25.7 to 37.3)	0.9621	2	9.4 (6.3 to 13.9)	5.7 (3.5 to 9.1)	0.0751
	Weekly or more	49.9 (43.2 to 56.6)	53.3 (47.1 to 59.4)	0.4999	≥3	5.5 (3.1 to 9.4)	3.7 (2.4 to 5.6)	0.25

© 2020 Ueda P et al. *JAMA Network Open.*

eTable 6. Trends in Sexual Frequency in the Last Year Among Men in the Total Age Range and by Age Group

		2000/2002	2004/2006	2008/2010	2012/2014	2016/2018	
Age group		**% (95% CI)**	**% (95% CI)**	**% (95% CI)**	**% (95% CI)**	**% (95% CI)**	**Age-adjusted OR for study period**
**18-44 y**	Not at all	9.5 (7.5 to 12)	10.3 (8.3 to 12.6)	8.7 (6.7 to 11.2)	14.9 (12 to 18.3)	16.5 (13 to 20.7)	1.18 (1.08 to 1.29)
	Once or twice/year	8 (6.4 to 9.8)	8.3 (6.3 to 11)	9.2 (7.1 to 11.7)	7.9 (5.8 to 10.7)	9.1 (6.8 to 12.1)	1.02 (0.93 to 1.12)
	1-3 times/month	22.1 (19.7 to 24.8)	23.5 (20.3 to 27)	24.5 (21.2 to 28)	23.9 (20.8 to 27.2)	27.7 (24.2 to 31.4)	1.07 (1.01 to 1.13)
	Weekly or more	60.4 (57.1 to 63.6)	57.9 (54 to 61.7)	57.7 (53.6 to 61.6)	53.3 (49.6 to 57)	46.7 (42.3 to 51.2)	0.89 (0.84 to 0.93)
**18-24 y**	Not at all	18.9 (13.8 to 25.5)	16.6 (11.9 to 22.7)	12 (7.7 to 18.3)	29.3 (22.2 to 37.6)	30.9 (22.7 to 40.5)	1.2 (1.04 to 1.39)
	Once or twice/year	13.1 (8.8 to 19.1)	16.1 (10.2 to 24.3)	18.2 (12.4 to 25.8)	13.1 (7.4 to 22.2)	8.9 (4.8 to 16)	0.91 (0.78 to 1.06)
	1-3 times/month	16.2 (11.5 to 22.3)	20.8 (15.6 to 27.3)	22 (15.5 to 30.2)	17.3 (11.8 to 24.7)	22.7 (16.4 to 30.6)	1.07 (0.94 to 1.21)
	Weekly or more	51.8 (44.5 to 59)	46.5 (38.8 to 54.4)	47.8 (38.9 to 56.9)	40.2 (31.3 to 49.9)	37.4 (28.6 to 47.2)	0.88 (0.79 to 0.99)
**25-34 y**	Not at all	7 (4.6 to 10.5)	8.1 (5.3 to 12)	7 (4.2 to 11.2)	12.3 (8.7 to 17)	14.1 (9.7 to 20)	1.23 (1.07 to 1.42)
	Once or twice/year	7 (4.8 to 10.2)	7.2 (4.7 to 10.8)	4.5 (2.6 to 7.5)	5.7 (3.6 to 8.9)	7.5 (4.8 to 11.6)	0.98 (0.84 to 1.14)
	1-3 times/month	20.6 (16.6 to 25.3)	19.9 (15.7 to 24.9)	22.4 (17.8 to 27.7)	21.4 (17.1 to 26.4)	28.1 (22.6 to 34.4)	1.09 (0.99 to 1.19)
	Weekly or more	65.3 (60 to 70.3)	64.9 (59 to 70.4)	66.2 (60.1 to 71.8)	60.6 (55.3 to 65.6)	50.3 (44 to 56.5)	0.87 (0.81 to 0.94)
**35-44 y**	Not at all	5.9 (4.1 to 8.4)	7.9 (5.8 to 10.5)	8.2 (5 to 13.2)	7.7 (5.2 to 11.2)	8 (5 to 12.7)	1.07 (0.94 to 1.22)
	Once or twice/year	5.6 (3.6 to 8.6)	3.8 (2.2 to 6.4)	7.8 (4.8 to 12.3)	6.9 (4.1 to 11.2)	11.1 (6.8 to 17.6)	1.24 (1.04 to 1.48)
	1-3 times/month	27.5 (23.6 to 31.7)	29.4 (23.5 to 36)	28.1 (22.8 to 34.2)	31.8 (25.8 to 38.4)	31 (25 to 37.6)	1.05 (0.97 to 1.14)
	Weekly or more	61.1 (56.4 to 65.5)	59 (52.5 to 65.1)	55.9 (49.8 to 61.9)	53.7 (47.3 to 60)	49.9 (43.2 to 56.6)	0.89 (0.83 to 0.96)

© 2020 Ueda P et al. *JAMA Network Open.*

eTable 7. Trends in the Number of Sexual Partners in the Last Year Among Men in the Total Age Range and by Age Group

		2000/2002	2004/2006	2008/2010	2012/2014	2016/2018	
		**% (95% CI)**	**% (95% CI)**	**% (95% CI)**	**% (95% CI)**	**% (95% CI)**	**Age-adjusted OR for study period**
**18-44 y**	0	10.1 (8.2 to 12.5)	10 (8 to 12.4)	9.6 (7.5 to 12.2)	13.6 (10.8 to 16.8)	16.4 (12.9 to 20.7)	1.15 (1.06 to 1.25)
	1	64.3 (61.1 to 67.3)	65.3 (61.5 to 69)	63.5 (59.2 to 67.5)	63.7 (59.5 to 67.7)	57.5 (52.2 to 62.6)	0.95 (0.9 to 1)
	2	9.3 (7.7 to 11.2)	9.6 (7.8 to 11.8)	10.1 (7.7 to 13.1)	7.9 (5.9 to 10.5)	11.6 (9.1 to 14.7)	1.02 (0.94 to 1.11)
	3 or more	16.3 (14 to 18.9)	15 (12.5 to 18)	16.9 (14.1 to 20.1)	14.8 (12.1 to 18)	14.5 (11.3 to 18.4)	0.96 (0.9 to 1.03)
**18-24 y**	0	18.3 (13.2 to 24.7)	15.9 (11.2 to 22)	14.1 (9.3 to 20.8)	27.6 (20.6 to 35.9)	32.1 (23.7 to 41.7)	1.23 (1.06 to 1.42)
	1	44.2 (37.5 to 51.1)	44.4 (36 to 53.2)	44.8 (36.4 to 53.6)	37.7 (29.7 to 46.3)	30 (23 to 38.1)	0.88 (0.8 to 0.98)
	2	9.3 (5.8 to 14.5)	16.3 (11.6 to 22.6)	17.2 (11.2 to 25.5)	13 (7.4 to 21.7)	14.7 (9.5 to 22.1)	1.08 (0.93 to 1.24)
	3 or more	28.3 (22.6 to 34.8)	23.3 (17.4 to 30.6)	23.9 (16.9 to 32.6)	21.7 (15.3 to 30)	23.2 (16.4 to 31.8)	0.93 (0.83 to 1.05)
**25-34 y**	0	7.8 (5.5 to 11)	9.3 (6.3 to 13.5)	7.9 (5.1 to 12.2)	11.4 (8.1 to 15.8)	13.6 (9.2 to 19.7)	1.16 (1.01 to 1.33)
	1	66.1 (60.8 to 71)	66.4 (60.3 to 72)	65.1 (58.5 to 71.1)	66.9 (60.7 to 72.5)	58.0 (50.7 to 65)	0.94 (0.86 to 1.02)
	2	12.2 (9.1 to 16.1)	8.3 (6.1 to 11.3)	8.6 (5.3 to 13.7)	7.1 (4.6 to 10.7)	11.6 (7.4 to 17.5)	0.95 (0.82 to 1.1)
	3 or more	13.9 (10.9 to 17.6)	16 (11.8 to 21.3)	18.4 (14.4 to 23.2)	14.6 (10.9 to 19.4)	16.8 (11.8 to 23.3)	1.04 (0.94 to 1.16)
**35-44 y**	0	7.3 (5.2 to 10.1)	6.4 (4.5 to 8.9)	8.3 (5.4 to 12.5)	6.5 (4.4 to 9.6)	7.8 (4.9 to 12.4)	1.02 (0.89 to 1.16)
	1	74.9 (70.6 to 78.8)	79.7 (76 to 83)	74.5 (68.7 to 79.5)	77.8 (72.1 to 82.5)	77.3 (71 to 82.5)	1.02 (0.94 to 1.11)
	2	6.6 (4.7 to 9.1)	6 (4 to 8.9)	6.6 (4.3 to 10.1)	5.5 (3.4 to 8.8)	9.4 (6.3 to 13.9)	1.07 (0.93 to 1.23)
	3 or more	11.2 (8.6 to 14.6)	7.9 (5.7 to 10.8)	10.6 (7.6 to 14.6)	10.2 (6.9 to 15)	5.5 (3.1 to 9.5)	0.9 (0.8 to 1.02)

© 2020 Ueda P et al. *JAMA Network Open.*

eTable 8. Trends in Sexual Frequency in Last Year Among Women in the Total Age Range and by Age Group

		2000/2002	2004/2006	2008/2010	2012/2014	2016/2018	
		**% (95% CI)**	**% (95% CI)**	**% (95% CI)**	**% (95% CI)**	**% (95% CI)**	**Age-adjusted OR for study period**
**18-44 y**	Not at all	10.1 (8.3 to 12.2)	9.8 (7.8 to 12.2)	9.7 (7.7 to 12.2)	10.4 (8.3 to 13.1)	12.6 (9.9 to 15.8)	1.05 (0.97 to 1.14)
	Once or twice/year	7.8 (6.2 to 9.8)	6.3 (5 to 8)	8 (6.2 to 10.3)	7.2 (5.5 to 9.3)	7.9 (6 to 10.4)	1.01 (0.93 to 1.11)
	1-3 times/month	24.8 (22.2 to 27.6)	27.7 (24.8 to 30.7)	27.9 (24.9 to 31.1)	27.1 (24.1 to 30.4)	26.2 (23 to 29.7)	1.02 (0.97 to 1.07)
	Weekly or more	57.3 (54.3 to 60.2)	56.2 (53 to 59.3)	54.4 (51 to 57.8)	55.2 (51.7 to 58.7)	53.3 (49.2 to 57.4)	0.96 (0.92 to 1.01)
**18-24 y**	Not at all	15.1 (10.6 to 20.9)	20.6 (14.6 to 28.4)	12.6 (7.8 to 19.6)	18.7 (12.2 to 27.6)	19.1 (12.7 to 27.7)	1.03 (0.89 to 1.18)
	Once or twice/year	9.4 (5.7 to 15)	8 (4.8 to 13)	13.4 (8.4 to 20.7)	10.4 (5.7 to 18.2)	10.1 (6 to 16.6)	1.04 (0.88 to 1.24)
	1-3 times/month	21.2 (15.7 to 27.9)	19.2 (13.6 to 26.5)	20.9 (15.4 to 27.8)	24.2 (17.6 to 32.3)	18.6 (12.8 to 26.2)	1 (0.88 to 1.13)
	Weekly or more	54.4 (47 to 61.6)	52.1 (44.1 to 60)	53.2 (45.3 to 60.8)	46.7 (37.7 to 56)	52.1 (42.9 to 61.2)	0.97 (0.87 to 1.07)
**25-34 y**	Not at all	7 (5.1 to 9.5)	5.7 (3.6 to 9)	7.4 (4.7 to 11.4)	7.2 (4.6 to 11)	12.6 (8.5 to 18.3)	1.17 (1.01 to 1.35)
	Once or twice/year	4.7 (3.1 to 7.1)	5.7 (3.8 to 8.5)	6 (3.9 to 9.1)	5.6 (3.6 to 8.6)	7.4 (5.1 to 10.6)	1.1 (0.96 to 1.25)
	1-3 times/month	21.8 (18.2 to 25.9)	26.2 (22.1 to 30.8)	29.4 (24.4 to 34.8)	25.9 (21.1 to 31.3)	25.9 (21 to 31.4)	1.05 (0.97 to 1.13)
	Weekly or more	66.4 (62.2 to 70.4)	62.3 (57.2 to 67.1)	57.3 (51.8 to 62.5)	61.3 (55.5 to 66.8)	54.2 (48.1 to 60.1)	0.9 (0.84 to 0.96)
**35-44 y**	Not at all	10 (7.6 to 13.2)	7.4 (5 to 10.9)	10.4 (7.5 to 14.4)	9.4 (7.1 to 12.2)	8.5 (5.4 to 12.9)	0.99 (0.88 to 1.12)
	Once or twice/year	9.7 (7.6 to 12.5)	5.9 (4.1 to 8.5)	7 (4.7 to 10.4)	7.1 (4.6 to 10.7)	7.1 (4.6 to 10.6)	0.93 (0.82 to 1.05)
	1-3 times/month	29.5 (25.3 to 34.1)	34.1 (29.3 to 39.3)	30.3 (25.5 to 35.5)	29.9 (25.2 to 35.2)	31.2 (25.7 to 37.3)	1 (0.93 to 1.08)
	Weekly or more	50.7 (45.7 to 55.6)	52.5 (47.2 to 57.8)	52.3 (46.4 to 58.2)	53.6 (48 to 59.1)	53.3 (47.1 to 59.4)	1.02 (0.96 to 1.1)

© 2020 Ueda P et al. *JAMA Network Open.*

eTable 9. Trends in the Number of Sexual Partners in the Last Year Among Women in the Total Age Range and by Age Group

		2000/2002	2004/2006	2008/2010	2012/2014	2016/2018	
		**% (95% CI)**	**% (95% CI)**	**% (95% CI)**	**% (95% CI)**	**% (95% CI)**	**Age-adjusted OR for study period**
**18-44 y**	0	10.3 (8.5 to 12.5)	9.8 (7.9 to 12.2)	9.4 (7.4 to 11.9)	9.2 (7.1 to 11.8)	12 (9.6 to 14.9)	1.02 (0.94 to 1.1)
	1	76.1 (73.4 to 78.7)	76.7 (73.8 to 79.4)	73.4 (70.1 to 76.4)	75.5 (72.1 to 78.6)	74.2 (70.5 to 77.5)	0.97 (0.92 to 1.03)
	2	8.5 (6.9 to 10.4)	7.9 (6.4 to 9.7)	10.8 (8.6 to 13.6)	8.7 (6.6 to 11.3)	6.8 (4.9 to 9.2)	0.97 (0.9 to 1.06)
	3 or more	5 (3.9 to 6.5)	5.6 (4.4 to 7.1)	6.4 (4.9 to 8.3)	6.7 (5 to 8.8)	7.1 (5.3 to 9.5)	1.1 (1 to 1.2)
**18-24 y**	0	15.2 (10.8 to 21)	21 (14.9 to 28.7)	12.2 (7.4 to 19.5)	17.5 (11.4 to 26)	20.1 (13.6 to 28.6)	1.02 (0.89 to 1.18)
	1	61.3 (54.5 to 67.6)	54.8 (47.2 to 62.2)	53.3 (45.9 to 60.6)	52.7 (43.2 to 62)	59.7 (50.3 to 68.4)	0.97 (0.88 to 1.08)
	2	12.8 (8.9 to 18.2)	12.5 (8.4 to 18.2)	20.5 (14.3 to 28.5)	16.2 (10.2 to 24.8)	7.7 (4.3 to 13.5)	0.97 (0.85 to 1.11)
	3 or more	10.7 (7.3 to 15.4)	11.7 (7.9 to 16.8)	14 (9.6 to 19.9)	13.5 (8.6 to 20.6)	12.5 (7.8 to 19.6)	1.06 (0.92 to 1.22)
**25-34 y**	0	6.7 (4.9 to 9.3)	5.5 (3.4 to 8.6)	7.8 (5.1 to 11.7)	6.5 (4.1 to 10.1)	12.8 (8.7 to 18.4)	1.18 (1.02 to 1.37)
	1	79.6 (75.8 to 83)	81.6 (77.4 to 85.2)	74.8 (69.6 to 79.4)	79.2 (74.7 to 83)	72.7 (66.8 to 77.9)	0.91 (0.84 to 0.99)
	2	10.1 (7.5 to 13.5)	7.7 (5.4 to 10.9)	10.7 (7.4 to 15.1)	7.4 (5.1 to 10.7)	7.3 (4.6 to 11.3)	0.93 (0.82 to 1.05)
	3 or more	3.5 (2.3 to 5.3)	5.2 (3.6 to 7.5)	6.8 (4.7 to 9.6)	6.9 (4.7 to 10.1)	7.3 (4.8 to 10.9)	1.2 (1.05 to 1.36)
**35-44 y**	0	10.8 (8.3 to 14)	7.8 (5.4 to 11.2)	9.4 (6.5 to 13.5)	7.6 (5.5 to 10.3)	6.3 (4.4 to 9)	0.89 (0.8 to 0.99)
	1	81.3 (77.6 to 84.4)	84.2 (80 to 87.7)	82.9 (78.2 to 86.8)	83.4 (80.2 to 86.1)	84.3 (80 to 87.8)	1.04 (0.96 to 1.12)
	2	4.6 (3.2 to 6.6)	5.5 (3.7 to 8.2)	5.8 (3.9 to 8.4)	6.1 (4.1 to 8.9)	5.7 (3.5 to 9.2)	1.06 (0.93 to 1.21)
	3 or more	3.3 (1.9 to 5.6)	2.5 (1.4 to 4.5)	1.9 (1 to 3.4)	3 (1.7 to 5)	3.7 (2.4 to 5.7)	1.02 (0.84 to 1.24)

© 2020 Ueda P et al. *JAMA Network Open.*

eTable 10. Trends in Sexual Frequency Among Men and Women Aged 18-44 Years by Marital Status

			2000/2002	2004/2006	2008/2010	2012/2014	2016/2018	
			**% (95% CI)**	**% (95% CI)**	**% (95% CI)**	**% (95% CI)**	**% (95% CI)**	**Age-adjusted OR for study period**
**Men**	**Unmarried** (n=2615; weighted %, 59.6)	Not at all	16.2 (13 to 20.1)	16.7 (13.5 to 20.5)	13.1 (10.1 to 16.7)	21.9 (17.6 to 26.9)	24.4 (19.3 to 30.3)	1.14 (1.04 to 1.25)
		Once or twice/year	11.1 (8.7 to 14.1)	11.9 (8.7 to 16.1)	12.6 (9.6 to 16.2)	10.9 (7.8 to 15.2)	11.2 (8 to 15.6)	0.99 (0.9 to 1.1)
		1-3 times/month	20.1 (17 to 23.7)	21.5 (17.6 to 26)	22.5 (18.3 to 27.4)	18.7 (15.1 to 22.8)	23.6 (19.3 to 28.5)	1.02 (0.95 to 1.1)
		Weekly or more	52.5 (48.3 to 56.6)	49.9 (44.7 to 55)	51.8 (46 to 57.6)	48.5 (43.6 to 53.5)	40.9 (35.6 to 46.4)	0.91 (0.86 to 0.97)
	**Married** (n=1674; weighted %, 40.4)	Not at all	0.4 (0.1 to 1.8)	1.8 (0.8 to 4)	2.3 (0.9 to 5.7)	3.9 (2.1 to 7.2)	1.7 (0.6 to 4.6)	1.36 (1.09 to 1.69)
		Once or twice/year	3.7 (2.3 to 6)	3.7 (2.3 to 5.8)	4.2 (2.3 to 7.7)	3.2 (1.6 to 6.3)	5.2 (3 to 8.9)	1.05 (0.88 to 1.27)
		1-3 times/month	24.7 (20.8 to 29.2)	26.1 (21.4 to 31.4)	27.3 (22.5 to 32.7)	32.1 (26.2 to 38.7)	35.4 (29.7 to 41.5)	1.14 (1.05 to 1.23)
		Weekly or more	71.1 (66.3 to 75.5)	68.4 (63.2 to 73.2)	66.2 (60.2 to 71.7)	60.8 (54.2 to 67.1)	57.7 (51.3 to 63.9)	0.86 (0.79 to 0.93)
**Women**	**Unmarried** (n=3030; weighted %, 53.1)	Not at all	19.1 (15.8 to 23)	19 (15.2 to 23.4)	16.3 (12.8 to 20.5)	18 (14.3 to 22.3)	20.6 (16.3 to 25.7)	1.01 (0.92 to 1.1)
		Once or twice/year	10.8 (8.5 to 13.7)	10.2 (7.8 to 13.2)	11.8 (9 to 15.4)	8.7 (6.2 to 12.1)	9.7 (7.2 to 12.8)	0.96 (0.87 to 1.06)
		1-3 times/month	24.5 (21 to 28.4)	25.4 (21.5 to 29.7)	24.3 (20.7 to 28.3)	25.2 (21.5 to 29.4)	21.8 (17.9 to 26.3)	0.97 (0.91 to 1.04)
		Weekly or more	45.5 (41.6 to 49.6)	45.4 (40.9 to 50.1)	47.6 (42.8 to 52.4)	48.1 (43.1 to 53.1)	47.9 (42.8 to 53.1)	1.03 (0.97 to 1.09)
	**Married** (n=2183; weighted %, 46.9)	Not at all	1 (0.3 to 2.9)	0.5 (0.1 to 1.9)	1.8 (0.8 to 3.8)	1.3 (0.6 to 3)	1.3 (0.4 to 3.7)	1.14 (0.81 to 1.59)
		Once or twice/year	4.8 (3.2 to 7.2)	2.4 (1.5 to 4)	3.4 (1.8 to 6.2)	5.3 (3.3 to 8.5)	5.5 (2.9 to 10.1)	1.09 (0.9 to 1.32)
		1-3 times/month	25.1 (21.5 to 29.1)	30 (26 to 34.3)	32.2 (27.7 to 37.2)	29.5 (24.8 to 34.6)	32.4 (26.8 to 38.5)	1.07 (1 to 1.15)
		Weekly or more	69.1 (65 to 72.9)	67.1 (62.7 to 71.1)	62.6 (57.6 to 67.4)	63.9 (58.7 to 68.8)	60.9 (54.3 to 67.1)	0.92 (0.86 to 0.99)

© 2020 Ueda P et al. *JAMA Network Open.*

eTable 11. Trends in Number of Sexual Partners in Last Year Among Men and Women Aged 18-44 Years by Marital Status

			2000/2002	2004/2006	2008/2010	2012/2014	2016/2018	
			**% (95% CI)**	**% (95% CI)**	**% (95% CI)**	**% (95% CI)**	**% (95% CI)**	**Age-adjusted OR for study period**
**Men**	**Unmarried** (n=2643; weighted %, 55.6)	0	16.2 (13.1 to 19.8)	16.5 (13.3 to 20.3)	14.2 (11.2 to 17.9)	20.6 (16.4 to 25.4)	24.9 (19.8 to 30.8)	1.14 (1.05 to 1.25)
		1	43.9 (40.2 to 47.7)	43.3 (37.8 to 48.9)	44.9 (39.8 to 50.2)	43.5 (38.4 to 48.8)	38.1 (32.7 to 43.9)	0.96 (0.9 to 1.02)
		2	14 (11.3 to 17.3)	14.7 (12 to 17.8)	15.8 (12.1 to 20.4)	12.4 (9.3 to 16.4)	15.6 (12.1 to 19.8)	1.01 (0.92 to 1.1)
		3 or more	25.9 (22.4 to 29.8)	25.6 (21.2 to 30.5)	25 (20.7 to 29.9)	23.5 (19.4 to 28.1)	21.4 (16.6 to 27.1)	0.94 (0.87 to 1.02)
	**Married** (n=1727; weighted %, 44.4)	0	2.1 (1.1 to 3.9)	1.7 (0.7 to 4.1)	2.9 (1.4 to 5.9)	2.9 (1.5 to 5.7)	1.1 (0.3 to 3.4)	0.99 (0.8 to 1.24)
		1	91.1 (88 to 93.5)	93.4 (90.2 to 95.7)	90.2 (86.1 to 93.2)	94.3 (90.7 to 96.6)	92.4 (87.3 to 95.6)	1.05 (0.91 to 1.22)
		2	3 (1.8 to 5.2)	3.2 (1.8 to 5.6)	1.8 (0.8 to 4)	1.1 (0.4 to 3)	4.6 (2.2 to 9.3)	0.99 (0.76 to 1.29)
		3 or more	3.7 (2.2 to 6.1)	1.6 (0.8 to 3.3)	5.1 (3.1 to 8.3)	1.7 (0.6 to 4.6)	1.9 (0.8 to 4.9)	0.88 (0.71 to 1.1)
**Women**	**Unmarried** (n=3088; weighted %, 47.9)	0	19 (15.7 to 22.9)	18.7 (15.1 to 23.1)	15.9 (12.4 to 20.1)	16.1 (12.5 to 20.5)	19.9 (15.8 to 24.6)	0.99 (0.91 to 1.08)
		1	56.1 (52.1 to 60)	57 (51.9 to 61.9)	54.2 (49.2 to 59.1)	58.9 (53.9 to 63.8)	58.4 (53.1 to 63.5)	1.03 (0.97 to 1.09)
		2	15.8 (12.9 to 19.2)	13.9 (11.4 to 16.8)	18.7 (14.7 to 23.4)	13.9 (10.5 to 18)	10.7 (7.8 to 14.3)	0.93 (0.85 to 1.01)
		3 or more	9.1 (7.1 to 11.6)	10.3 (8 to 13.3)	11.3 (8.6 to 14.6)	11.1 (8.3 to 14.7)	11.1 (8.3 to 14.8)	1.05 (0.96 to 1.16)
	**Married** (n=2289; weighted %, 52.1)	0	1.9 (1 to 3.8)	1.2 (0.5 to 2.7)	1.8 (0.8 to 3.8)	1 (0.4 to 2.3)	1.1 (0.4 to 2.8)	0.86 (0.65 to 1.13)
		1	95.5 (93.1 to 97.1)	95.9 (93.8 to 97.3)	96 (93.7 to 97.5)	95 (92.3 to 96.8)	96 (92.9 to 97.8)	1 (0.85 to 1.17)
		2	1.4 (0.8 to 2.8)	2 (1.1 to 3.7)	1.6 (0.8 to 3.2)	2.6 (1.4 to 4.9)	1.4 (0.4 to 4.6)	1.06 (0.84 to 1.33)
		3 or more	1.1 (0.4 to 2.8)	0.9 (0.3 to 2.4)	0.6 (0.2 to 1.9)	1.4 (0.5 to 3.7)	1.6 (0.7 to 3.6)	1.14 (0.81 to 1.61)

© 2020 Ueda P et al. *JAMA Network Open.*

eTable 12. Association Between Sociodemographic and Behavioral Variables and No Sexual Partners in the Last Year

	Men			Women		
	% (95% CI)	*Age-adjusted OR*	*n (unweighted/ weighted)*	*% (95% CI)*	*Age-adjusted OR*	*n (unweighted/ weighted)*
**Race**						
White	11.2 (9.9 to 12.6)	1 (ref)	3250/3502	9.5 (8.4 to 10.7)	1 (ref)	3736/3931
Black	9.9 (7.4 to 13.2)	0.77 (0.54 to 1.11)	577/617	11.7 (9.4 to 14.6)	1.25 (0.94 to 1.67)	1020/900.2
Other	16 (12.4 to 20.3)	1.39 (1.01 to 1.92)	545/647	11.5 (8.8 to 15)	1.2 (0.86 to 1.68)	621/695.9
**Sexual orientation**[a]						
Heterosexual	12.7 (11 to 14.6)	1 (ref)	2114/2344	10 (8.6 to 11.6)	1 (ref)	2505/2626
Gay/lesbian/bisexual	15.9 (9.3 to 25.9)	1.19 (0.62 to 2.29)	108/100.6	10.8 (6.9 to 16.6)	0.93 (0.55 to 1.57)	209/198.8
**Religion**						
None	12.7 (10.5 to 15.3)	1 (ref)	1194/1273	9.4 (7.5 to 11.6)	1 (ref)	1055/1079
Christian	10.4 (9.2 to 11.9)	0.91 (0.7 to 1.17)	2895/3176	9.9 (8.8 to 11.1)	1.17 (0.91 to 1.51)	4009/4112
Non-Christian	19.9 (14.6 to 26.6)	1.9 (1.22 to 2.95)	268/297.8	14.9 (10.5 to 20.7)	1.75 (1.09 to 2.83)	298/321.1
**Education**						
High school or less	12.8 (11.3 to 14.4)	1 (ref)	2815/3167	10.7 (9.5 to 12.1)	1 (ref)	3420/3584
College or more	9.3 (7.8 to 11.1)	0.91 (0.71 to 1.17)	1554/1595	8.8 (7.4 to 10.4)	0.92 (0.73 to 1.15)	1950/1935
**Employment**						
Fulltime	7.8 (6.7 to 9)	1 (ref)	3149/3300	8.9 (7.7 to 10.3)	1 (ref)	2692/2670
Part-time	19 (14.9 to 23.9)	2.03 (1.45 to 2.83)	405/504	10.4 (8.1 to 13.2)	1.11 (0.81 to 1.51)	859/918.1
Student	28.3 (22.9 to 34.3)	2.91 (2.05 to 4.13)	297/379.4	23.9 (19.1 to 29.4)	2.53 (1.8 to 3.57)	386/431.6
Not working	15.8 (12.2 to 20.2)	2.04 (1.45 to 2.85)	452/516.7	6.8 (5.4 to 8.5)	0.75 (0.56 to 1)	1362/1422
**Annual income (USD)**[b]						
0-9,999	26 (19.5 to 33.9)	1 (ref)	212/262.3	12.9 (9.5 to 17.2)	1 (ref)	386/449.4
10,000-49,999	12.7 (8.3 to 18.8)	0.54 (0.29 to 1.01)	255/263.6	11.6 (7.9 to 16.8)	1.13 (0.64 to 1.99)	282/281
≥50,000	6.9 (3.6 to 13)	0.42 (0.17 to 1.03)	183/193.9	5.6 (2.6 to 12)	0.73 (0.31 to 1.74)	102/100.8
**Steady partner**[c]						

© 2020 Ueda P et al. *JAMA Network Open.*

No	30.7 (25.4 to 36.6)	1 (ref)	344/383.7			323/317.5
Yes	2.3 (1.1 to 5)	0.07 (0.03 to 0.16)	359/397.9	1.3 (0.6 to 2.7)	0.03 (0.01 to 0.06)	703/775.2
**Marital status**						
Married	2.2 (1.5 to 3)	1.00 (ref)	1959/1727	1.4 (1 to 2)	1.00 (ref)	2633/2289
Previously married	9.9 (7.3 to 13.4)	5.06 (3.09 to 8.28)	435.8/516	12.5 (10.2 to 15.1)	9.62 (6.19 to 14.95)	728/930
Never married	19.8 (17.8 to 22)	10.01 (6.78 to 14.78)	2370/2127	19.8 (17.7 to 22.1)	20.29 (13.63 to 30.22)	2166/2158
**Region**						
Northeast	13.6 (11 to 16.7)	1 (ref)	693/787.6			913/976.6
Midwest	12 (9.7 to 14.9)	0.85 (0.6 to 1.2)	1086/1134	8.5 (6.7 to 10.8)	0.71 (0.49 to 1.01)	1250/1234
South	9.9 (8.1 to 11.9)	0.7 (0.51 to 0.96)	1520/1647	9.6 (8.1 to 11.4)	0.81 (0.59 to 1.11)	1974/1972
West	12.4 (10.2 to 15.1)	0.86 (0.62 to 1.19)	1073/1198	11.3 (9.3 to 13.6)	0.95 (0.68 to 1.33)	1240/1345
**Residence**						
City >250,000.	12.2 (9.8 to 15)	1 (ref)	896/925.6	13.8 (11.4 to 16.6)	1 (ref)	1066/1072
City 50-250,000	11.3 (8.9 to 14.2)	0.95 (0.67 to 1.35)	914/964.6	10.8 (8.8 to 13.1)	0.76 (0.56 to 1.04)	1164/1167
Suburbs/small city	12.4 (10.7 to 14.3)	1.08 (0.81 to 1.43)	1933/2205	9.5 (8.1 to 11.1)	0.66 (0.5 to 0.88)	2331/2456
Rural	9 (6.7 to 11.9)	0.8 (0.54 to 1.19)	629/670.5	6.2 (4.6 to 8.3)	0.42 (0.28 to 0.62)	816/832.4
**Porn use in last year**						
No	15.2 (12.8 to 17.8)	1.00 (ref)	1434/1312	13.1 (11.6 to 14.8)	1.00 (ref)	2592/2466
Yes	10.3 (8.6 to 12.3)	0.5 (0.38 to 0.67)	1559/1437	4 (2.7 to 5.8)	0.24 (0.15 to 0.37)	909.7/927
**Hours of internet use per week[d]**						
0-5	9.5 (7.5 to 12)	1.00 (ref)	1075/979	9.8 (8 to 11.9)	1.00 (ref)	1496/1433
≥5 to <15	10 (7.7 to 13)	0.98 (0.66 to 1.43)	942.7/861	12.6 (10.3 to 15.4)	1.29 (0.94 to 1.78)	971.8/944
≥15	15.1 (12.1 to 18.7)	1.47 (1.01 to 2.16)	724.1/659	12 (9.3 to 15.3)	1.23 (0.83 to 1.81)	675.7/654
**Hours worked per week**						
0	21.3 (18 to 25)	1.00 (ref)	887.3/739	10.7 (9 to 12.7)	1.00 (ref)	1822/1720
1-20	18 (12.4 to 25.3)	0.81 (0.5 to 1.33)	233.6/190	10.9 (7.7 to 15.2)	0.99 (0.64 to 1.52)	436.7/403
21-40	10.6 (8.9 to 12.7)	0.53 (0.4 to 0.71)	1647/1512	9.1 (7.7 to 10.7)	0.87 (0.68 to 1.13)	2146/2111
41-60	6.7 (5.3 to 8.4)	0.37 (0.26 to 0.51)	1268/1224	8.8 (6.8 to 11.3)	0.87 (0.62 to 1.22)	768.9/785
≥60	6.3 (4.4 to 8.9)	0.34 (0.22 to 0.54)	641.2/616	10.8 (6.9 to 16.6)	1.18 (0.7 to 1.98)	239.5/247

© 2020 Ueda P et al. *JAMA Network Open.*

Age-adjusted odds ratios were calculated using logistic regression model using 3 or more sexual partners as the binary outcome variable (yes=1, no=0) and age and survey period as independent variables. Analyses included all participants who had been asked the question regarding the variable of interest. Missing variables were excluded by variable. For men, missing values were religion (n=15), sexual orientation (n=19), annual income (n=8), education (n=3), employment (n=69), stable relation (n=3), marital status (n=2), porn use (n=14), hours of internet use per week (n=37), hours worked per week (n=91). For women, missing values were religion (n=15), sexual orientation (n=33), education (n=7), annual income (n=12), stable relation (n=3), employment (n=78), porn use (n=12), hours of internet use per week (n=66), hours worked per week (n=111).

a. Using surveys of 2008-2018.
b. Using surveys of 2016-2018.
c. Using surveys of 2012-2018.
d. Not including the survey of 2008.

© 2020 Ueda P et al. *JAMA Network Open.*

eTable 13. Association Between Sociodemographic and Behavioral Variables and ≥Weekly Sex in the Last Year

	Men			Women		
	% (95% CI)	*Age-adjusted OR*	*n (unweighted/ weighted)*	*% (95% CI)*	*Age-adjusted OR*	*n (unweighted/ weighted)*
**Race**						
White	55.9 (53.9 to 57.8)	1 (ref)	3185/3429	56.8 (54.9 to 58.6)	1 (ref)	3628/3794
Black	58.1 (53.2 to 62.8)	1.15 (0.93 to 1.42)	573/611.2	51.9 (48.2 to 55.6)	0.82 (0.7 to 0.98)	989/876.9
Other	53.9 (48.7 to 58.9)	0.97 (0.77 to 1.21)	533/635	53.4 (48.8 to 57.8)	0.88 (0.72 to 1.07)	596/670.6
**Sexual orientation**[a]						
Heterosexual	53.3 (50.8 to 55.8)	1 (ref)	2079/2305	54.9 (52.6 to 57.2)	1 (ref)	2419/2520
Gay/lesbian/bisexual	42.9 (32.8 to 53.7)	0.68 (0.43 to 1.08)	106/98.1	52 (44.7 to 59.2)	0.90 (0.65 to 1.23)	206/196.2
**Religion**						
None	56.9 (53.6 to 60.1)	1 (ref)	1178/1257	58.3 (54.8 to 61.7)	1 (ref)	1037/1053
Christian	56.5 (54.3 to 58.6)	0.92 (0.79 to 1.07)	2841/3115	55.4 (53.6 to 57.1)	0.88 (0.75 to 1.04)	3877/3971
Non-Christian	44.7 (37.9 to 51.8)	0.58 (0.43 to 0.79)	257/284.5	48.6 (42.3 to 54.9)	0.67 (0.50 to 0.89)	287/307.1
**Education**						
High school or less	56.9 (54.7 to 59)	1 (ref)	2773/3122	56.2 (54.3 to 58.1)	1 (ref)	3325/3478
College or more	53.8 (51.1 to 56.5)	0.82 (0.71 to 0.95)	1515/1549	54.2 (51.6 to 56.8)	0.94 (0.82 to 1.08)	1881/1856
**Employment**						
Fulltime	60.2 (58.3 to 62.1)	1 (ref)	3082/3223	56 (53.9 to 58.1)	1 (ref)	2620/2589
Part-time	44.3 (38.6 to 50.2)	0.54 (0.42 to 0.7)	400/498.7	55.4 (51.5 to 59.3)	0.95 (0.8 to 1.14)	831/883.5
Student	35.2 (29.7 to 41.3)	0.38 (0.28 to 0.5)	296/379	46.1 (40.3 to 52.1)	0.61 (0.46 to 0.8)	381/426.7
Not working	55.2 (49.8 to 60.5)	0.82 (0.65 to 1.04)	444/509.4	59.1 (56 to 62.1)	1.14 (0.98 to 1.33)	1306/1359
**Annual income (USD)**[b]						
0-9,999	44.5 (37 to 52.3)	1 (ref)	210/259.8	55.6 (50.2 to 60.9)	1 (ref)	372/434.2
10,000-49,999	46 (39.1 to 53)	0.96 (0.62 to 1.48)	253/262.1	51.5 (44.8 to 58)	0.82 (0.58 to 1.16)	277/275.3
≥50,000	51.8 (44.5 to 59)	1.07 (0.67 to 1.69)	179/187.8	51.3 (40.4 to 62)	0.79 (0.47 to 1.31)	102/101.9
**Steady partner**[c]						
No	29.7 (24.5 to 35.4)	1 (ref)	339/382.4	32.9 (27 to 39.4)	1 (ref)	316/311.5
Yes	59 (53.3 to 64.5)	3.46 (2.4 to 5)	346/379.6	62.8 (58.4 to 67.1)	3.88 (2.7 to 5.57)	669/734.8

© 2020 Ueda P et al. *JAMA Network Open.*

**Marital status**						
Married	65.9 (63.4 to 68.4)	1.00 (ref)	1891/39.03	65.4 (63.3 to 67.6)	1.00 (ref)	2504/2183
Previously married	57.3 (52.2 to 62.3)	0.69 (0.54 to 0.87)	432.8/510	50.5 (46.8 to 54.2)	0.56 (0.47 to 0.67)	709.8/905
Never married	47.5 (45 to 50.1)	0.43 (0.36 to 0.51)	2351/2105	45.6 (43 to 48.1)	0.34 (0.29 to 0.39)	2127/2125
**Region**						
Northeast	55.5 (51 to 59.8)	1 (ref)	676/768.4	53.5 (49.6 to 57.4)	1 (ref)	866/924.3
Midwest	53.1 (49.5 to 56.6)	0.91 (0.72 to 1.15)	1072/1117	57.1 (54 to 60.1)	1.15 (0.94 to 1.4)	1218/1198
South	58.8 (56.1 to 61.5)	1.15 (0.93 to 1.43)	1497/1621	55.2 (52.7 to 57.7)	1.07 (0.89 to 1.29)	1919/1910
West	54.7 (51.1 to 58.3)	0.99 (0.79 to 1.26)	1046/1169	56 (52.9 to 59)	1.11 (0.9 to 1.35)	1210/1309
**Residence**						
City >250,000.	53.1 (49.2 to 56.9)	1 (ref)	875/903.4	52.7 (49.4 to 56.1)	1 (ref)	1033/1034
City 50-250,000	53.7 (50 to 57.3)	1.01 (0.82 to 1.24)	891/939.1	54.1 (50.8 to 57.4)	1.06 (0.88 to 1.28)	1124/1121
Suburbs/small city	56.2 (53.6 to 58.7)	1.10 (0.92 to 1.32)	1904/2169	55.4 (53.1 to 57.6)	1.11 (0.94 to 1.31)	2256/2377
Rural	61.8 (57.3 to 66.1)	1.36 (1.07 to 1.73)	621/663.6	61.6 (57.8 to 65.3)	1.43 (1.16 to 1.76)	800/808.9
**Porn use in last year**						
No	52.4 (49.2 to 55.6)	1.00 (ref)	1390/1273	50.4 (48.2 to 52.7)	1.00 (ref)	2478/2371
Yes	55.9 (52.9 to 59)	1.26 (1.05 to 1.52)	1543/1425	64.6 (61 to 68.1)	1.82 (1.51 to 2.19)	899.4/917
**Hours of internet use per week[d]**						
0-5	58.2 (54.7 to 61.5)	1.00 (ref)	1057/960	57.5 (54.5 to 60.4)	1.00 (ref)	1448/1389
≥5 to <15	56.7 (52.7 to 60.6)	1 (0.81 to 1.24)	930.2/849	54.4 (50.7 to 58.1)	0.88 (0.72 to 1.07)	944.7/920
≥15	49.2 (44.7 to 53.7)	0.78 (0.61 to 1)	709.3/648	48.9 (44.5 to 53.3)	0.7 (0.56 to 0.89)	664.7/642
**Hours worked per week**						
0	46.6 (42.5 to 50.7)	1.00 (ref)	879.6/730	56 (53.2 to 58.7)	1.00 (ref)	1755/1661
1-20	46.5 (38.3 to 54.8)	0.99 (0.68 to 1.46)	231.2/187	54.5 (48.6 to 60.2)	0.94 (0.72 to 1.22)	419.8/390
21-40	54.7 (51.8 to 57.6)	1.33 (1.09 to 1.63)	1613/1481	56.6 (54.2 to 59)	1.03 (0.88 to 1.2)	2063/2040
41-60	62.8 (59.5 to 65.9)	1.78 (1.43 to 2.23)	1249/1207	55.7 (51.8 to 59.5)	1 (0.82 to 1.21)	765.1/777
≥60	62.5 (57.9 to 66.9)	1.8 (1.39 to 2.32)	614.8/596	51.4 (43.6 to 59)	0.84 (0.6 to 1.18)	225.6/237

Age-adjusted odds ratios were calculated using logistic regression model using weekly sex as the binary outcome variable (yes=1, no=0) and age as a continuous independent variable. Analyses included all participants who had been asked the question regarding the variable of interest. Missing

© 2020 Ueda P et al. *JAMA Network Open.*

variables were excluded by variable. For men, missing values were religion (n=15), sexual orientation (n=15). annual income (n=8), education (n=3), employment (n=69), stable relation (n=3), marital status (n=2), porn use (n=12), hours of internet use per week (n=35), hours worked per week (n=90). For women, missing values were religion (n=12), sexual orientation (n=25), education (n=7), annual income (n=10), stable relation (n=1), employment (n=75), porn use (n=12), hours of internet use per week (n=64), hours worked per week (n=108).

a. Using surveys of 2008-2018.
b. Using surveys of 2016-2018.
c. Using surveys of 2012-2018.
d. Not including the survey of 2008.

eTable 14. Association Between Sociodemographic and Behavioral Variables and Having 3 or More Sexual Partners in the Last Year

	Men			Women		
	% (95% CI)	*Age-adjusted OR*	*n (unweighted/ weighted)*	*% (95% CI)*	*Age-adjusted OR*	*n (unweighted/ weighted)*
**Race**						
White	13.7 (12.4 to 15.2)	1 (ref)	3250/3502	6 (5.1 to 6.9)	1 (ref)	3736/3931
Black	26.4 (22.4 to 31)	2.19 (1.7 to 2.83)	577/617	7.6 (6.1 to 9.6)	1.25 (0.93 to 1.69)	1020/900.2
Other	15 (11.7 to 19.1)	1.03 (0.75 to 1.42)	545/647	4.3 (2.9 to 6.4)	0.65 (0.42 to 1)	621/695.9
**Sexual orientation**[a]						
Heterosexual	14.6 (12.8 to 16.6)	1 (ref)	2114/2344	5.9 (4.9 to 7.1)	1 (ref)	2505/2626
Gay/lesbian/bisexual	35.4 (26.1 to 45.9)	3.39 (2.08 to 5.54)	108/100.6	16.8 (11.9 to 23.3)	2.69 (1.71 to 4.24)	209/198.8
**Religion**						
None	18.1 (15.6 to 20.9)	1 (ref)	1194/1273	9.5 (7.7 to 11.6)	1 (ref)	1055/1079
Christian	14.5 (13.1 to 16)	0.82 (0.66 to 1.01)	2895/3176	5 (4.3 to 5.8)	0.6 (0.45 to 0.79)	4009/4112
Non-Christian	15.6 (11.1 to 21.4)	0.89 (0.57 to 1.37)	268/297.8	7.8 (4.8 to 12.4)	0.87 (0.5 to 1.52)	298/321.1
**Education**						
High school or less	17 (15.4 to 18.7)	1 (ref)	2815/3167	6.9 (6.1 to 7.9)	1 (ref)	3420/3584
College or more	12.7 (10.6 to 15.1)	0.9 (0.71 to 1.15)	1554/1595	4.4 (3.5 to 5.5)	0.84 (0.63 to 1.12)	1950/1935
**Employment**						
Fulltime	14.2 (12.9 to 15.6)	1 (ref)	3149/3300	5.8 (4.9 to 6.8)	1 (ref)	2692/2670
Part-time	19.7 (15.7 to 24.4)	0.96 (0.69 to 1.32)	405/504	6.5 (4.9 to 8.5)	0.9 (0.65 to 1.26)	859/918.1
Student	18.7 (14.3 to 24)	0.72 (0.5 to 1.02)	297/379.4	8.6 (6.1 to 12.2)	0.74 (0.48 to 1.16)	386/431.6
Not working	18.5 (14.6 to 23.2)	1.18 (0.87 to 1.6)	452/516.7	4.7 (3.6 to 6)	0.81 (0.59 to 1.11)	1362/1422
**Annual income (USD)**[b]						
0-9,999	19.5 (14.1 to 26.2)	1 (ref)	212/262.3	8.6 (5.8 to 12.7)	1 (ref)	386/449.4
10,000-49,999	11.7 (7.6 to 17.6)	0.74 (0.43 to 1.29)	255/263.6	6.5 (4.2 to 9.8)	0.92 (0.49 to 1.74)	282/281
≥50,000	8.8 (5.3 to 14.2)	0.83 (0.42 to 1.63)	183/193.9	3.8 (2 to 6.9)	0.73 (0.34 to 1.57)	102/100.8
**Steady partner**[c]						
No	30.4 (25.1 to 36.2)	1 (ref)	344/383.7	13.5 (9.8 to 18.2)	1 (ref)	323/317.5
Yes	6.1 (3.9 to 9.4)	0.15 (0.08 to 0.28)	359/397.9	4.4 (3 to 6.3)	0.39 (0.21 to 0.7)	703/775.2

© 2020 Ueda P et al. *JAMA Network Open.*

**Marital status**						
Married	2.8 (2.1 to 3.8)	1.00 (ref)	1959/1727	1.1 (0.7 to 1.7)	1.00 (ref)	2633/2289
Previously married	21.5 (17.6 to 26.1)	9.49 (6.37 to 14.13)	435.8/516	10.9 (8.9 to 13.1)	12.21 (7.48 to 19.94)	728/930
Never married	25 (22.9 to 27.2)	10.41 (7.46 to 14.53)	2370/2127	10.4 (9 to 12)	6.2 (3.73 to 10.28)	2166/2158
**Region**						
Northeast	16.3 (13.3 to 19.8)	1 (ref)	693/787.6	6.2 (4.5 to 8.4)	1 (ref)	913/976.6
Midwest	13.4 (11.1 to 15.9)	0.76 (0.55 to 1.05)	1086/1134	5.8 (4.5 to 7.5)	0.88 (0.58 to 1.34)	1250/1234
South	16.4 (14.3 to 18.7)	1.01 (0.76 to 1.35)	1520/1647	5.6 (4.6 to 6.7)	0.83 (0.56 to 1.23)	1974/1972
West	16 (13.4 to 19)	0.93 (0.67 to 1.28)	1073/1198	6.8 (5.3 to 8.6)	0.99 (0.64 to 1.51)	1240/1345
**Residence**						
City >250,000.	18.9 (16.3 to 21.8)	1 (ref)	896/925.6	7.3 (5.5 to 9.5)	1 (ref)	1066/1072
City 50-250,000	15.8 (13.4 to 18.6)	0.82 (0.62 to 1.07)	914/964.6	7.5 (5.9 to 9.4)	1.07 (0.72 to 1.58)	1164/1167
Suburbs/small city	14.6 (12.8 to 16.6)	0.75 (0.59 to 0.95)	1933/2205	5.3 (4.4 to 6.4)	0.76 (0.53 to 1.09)	2331/2456
Rural	13.6 (10.6 to 17.5)	0.72 (0.51 to 1.02)	629/670.5	4.6 (3.4 to 6.1)	0.67 (0.43 to 1.02)	816/832.4
**Porn use in last year**						
No	7.4 (6.1 to 9.1)	1.00 (ref)	1434/1312	4.3 (3.5 to 5.3)	1.00 (ref)	2592/2466
Yes	22.4 (20 to 25)	3.26 (2.52 to 4.23)	1559/1437	11.7 (9.6 to 14.2)	2.56 (1.89 to 3.46)	909.7/927
**Hours of internet use per week[d]**						
0-5	14.8 (12.3 to 17.6)	1.00 (ref)	1075/979	4.2 (3.2 to 5.4)	1.00 (ref)	1496/1433
≥5 to <15	15.9 (13.2 to 19)	1.03 (0.76 to 1.4)	942.7/861	6.6 (4.9 to 8.7)	1.4 (0.92 to 2.12)	971.8/944
≥15	16.9 (13.7 to 20.7)	1.05 (0.75 to 1.48)	724.1/659	8.7 (6.7 to 11.3)	1.95 (1.26 to 3)	675.7/654
**Hours worked per week**						
0	18.8 (15.8 to 22.2)	1.00 (ref)	887.3/739	5.6 (4.5 to 6.8)	1.00 (ref)	1822/1720
1-20	18.9 (13.4 to 26.1)	1.02 (0.63 to 1.64)	233.6/190	5 (3.2 to 7.8)	1.1 (0.75 to 1.61)	436.7/403
21-40	15.8 (13.7 to 18.1)	1.05 (0.8 to 1.38)	1647/1512	6.3 (5.2 to 7.6)	1.35 (0.9 to 2.02)	2146/2111
41-60	14.2 (12.1 to 16.6)	1.06 (0.79 to 1.42)	1268/1224	4.8 (3.5 to 6.6)	1.39 (0.92 to 2.11)	768.9/785
≥60	12.7 (10.1 to 15.9)	0.97 (0.69 to 1.36)	641.2/616	8.2 (4.9 to 13.3)	1.39 (0.89 to 2.17)	239.5/247

Age-adjusted odds ratios were calculated using logistic regression model using 3 or more sexual partners as the binary outcome variable (yes=1, no=0) and age and survey period as independent variables. Analyses included all participants who had been asked the question regarding the

© 2020 Ueda P et al. *JAMA Network Open.*

variable of interest. Missing variables were excluded by variable. For men, missing values were religion (n=15), sexual orientation (n=19), annual income (n=8), education (n=3), employment (n=69), stable relation (n=3), marital status (n=2), porn use (n=14), hours of internet use per week (n=37), hours worked per week (n=91). For women, missing values were religion (n=15), sexual orientation (n=33), education (n=7), annual income (n=12), stable relation (n=3), employment (n=78), porn use (n=12), hours of internet use per week (n=66), hours worked per week (n=111).

a. Using surveys of 2008-2018.
b. Using surveys of 2016-2018.
c. Using surveys of 2012-2018.
d. Not including the survey of 2008.

© 2020 Ueda P et al. *JAMA Network Open.*

eTable 15. Trends in Sexual Inactivity in Last Year Among Men in Sociodemographic Subgroups

		2000/2002	2004/2006	2008/2010	2012/2014	2016/2018		
		**% (95% CI)**	**% (95% CI)**	**% (95% CI)**	**% (95% CI)**	**% (95% CI)**	**Age-adjusted OR for study period**	*p* for interaction
Race	White	10.6 (8.2 to 13.6)	10.4 (8.3 to 12.9)	8.3 (6 to 11.4)	13.4 (10.1 to 17.5)	16.9 (13 to 21.6)	1.14 (1.03 to 1.26)	
	Black	3 (1.1 to 7.5)	5.2 (2.3 to 11.5)	8.2 (3.6 to 17.7)	18.9 (11.4 to 29.8)	11.5 (6.6 to 19.3)	1.44 (1.17 to 1.76)	
	Other	8.9 (4.4 to 16.9)	13.2 (6.4 to 25.2)	11.1 (5.7 to 20.6)	17.6 (11.6 to 25.8)	21.2 (11.7 to 35.4)	1.32 (1.04 to 1.68)	0.204
Sexual orientation	Heterosexual	-	-	8.4 (6.4 to 11)	14.3 (11.3 to 17.9)	16.3 (12.7 to 20.7)	1.41 (1.16 to 1.71)	
	Gay/lesbian/bisexual	-	-	18.6 (6.7 to 42.4)	24.2 (13.6 to 39.3)	10.5 (3 to 30.9)	0.62 (0.29 to 1.33)	0.05
Religion	None	13.4 (8.7 to 20)	9.9 (5.9 to 16.2)	8.1 (5 to 12.7)	19.5 (13.6 to 27)	12.4 (8.5 to 17.8)	1.09 (0.94 to 1.27)	
	Christian	8.4 (6.4 to 11)	9.6 (7.3 to 12.4)	8.6 (6.3 to 11.5)	10.5 (7.9 to 13.7)	17.5 (12.8 to 23.6)	1.18 (1.06 to 1.32)	
	Non-Christian	8.2 (3.8 to 16.8)	19.2 (9.6 to 34.6)	15.3 (5.5 to 35.9)	36.6 (22.3 to 53.8)	25.6 (11.3 to 48.3)	1.35 (1.05 to 1.74)	0.177
Education	High school or less	10.3 (7.9 to 13.4)	11.5 (8.6 to 15.4)	7.9 (5.5 to 11.1)	16.6 (12.9 to 21.1)	18 (13.7 to 23.4)	1.17 (1.06 to 1.3)	
	College or more	7.1 (4.9 to 10.3)	8.3 (5.8 to 11.7)	10.6 (6.8 to 16.2)	11.6 (8.2 to 16.3)	13.4 (8.7 to 20)	1.21 (1.06 to 1.38)	0.691
Employment	Fulltime	5.8 (4.3 to 7.7)	7.4 (5.6 to 9.7)	7 (4.8 to 10.1)	8.1 (5.8 to 11.3)	11.6 (8.2 to 16.4)	1.18 (1.05 to 1.32)	
	Part-time	25.7 (17.2 to 36.5)	8.7 (3.8 to 18.8)	9.6 (5 to 17.5)	24.9 (15.4 to 37.7)	23.2 (13.5 to 36.8)	1.06 (0.85 to 1.32)	
	Student	19.8 (9.9 to 35.7)	32.3 (22.5 to 44)	26 (15 to 41.3)	38 (26.2 to 51.5)	22.7 (12.5 to 37.6)	1.06 (0.87 to 1.29)	
	Not working	12.7 (6.2 to 24.3)	13.3 (7.6 to 22.3)	7.1 (3.3 to 14.8)	20.8 (12.9 to 31.8)	29.2 (18.7 to 42.4)	1.33 (1.02 to 1.71)	0.882
Married	No	16.2 (13 to 20.1)	16.7 (13.5 to 20.5)	13.1 (10.1 to 16.7)	21.9 (17.6 to 26.9)	24.4 (19.3 to 30.3)	1.14 (1.04 to 1.25)	
	Yes	0.4 (0.1 to 1.8)	1.8 (0.8 to 4)	2.3 (0.9 to 5.7)	3.9 (2.1 to 7.2)	1.7 (0.6 to 4.6)	1.36 (1.09 to 1.69)	0.151
Region	Northeast	11 (7.3 to 16.1)	9.9 (5.9 to 16.1)	6.7 (2.7 to 15.4)	14.2 (9.2 to 21.2)	25.4 (16.2 to 37.5)	1.25 (1.03 to 1.51)	
	Midwest	13.3 (8.9 to 19.6)	9.3 (6.3 to 13.6)	8.5 (3.6 to 19)	12.2 (6.9 to 20.5)	13.9 (7.9 to 23.2)	1.03 (0.86 to 1.24)	
	South	5.6 (3.2 to 9.8)	10.1 (6.9 to 14.7)	7.4 (5.3 to 10.2)	14.3 (10.3 to 19.5)	14.7 (9 to 23)	1.27 (1.08 to 1.5)	
	West	8.8 (5.1 to 14.7)	11.5 (7.6 to 17)	11.5 (7.7 to 16.6)	18.4 (12.2 to 26.8)	15.9 (10.4 to 23.5)	1.19 (1.01 to 1.39)	0.697
Residence	City >250,000.	8.4 (4.9 to 14.1)	10.1 (6.6 to 15.3)	9.6 (5.4 to 16.4)	14.9 (9 to 23.6)	18.7 (12.6 to 26.8)	1.27 (1.07 to 1.51)	
	City 50-250,000	6.2 (3.3 to 11.4)	13.2 (7.9 to 21.1)	9.2 (5.6 to 14.7)	17.9 (11.9 to 26.1)	13.5 (7.4 to 23.2)	1.19 (0.99 to 1.43)	
	Suburbs/small city	10.8 (8.1 to 14.3)	9.2 (6.2 to 13.3)	8.5 (5.5 to 12.9)	15.9 (12 to 20.7)	17.1 (12.2 to 23.4)	1.18 (1.04 to 1.33)	
	Rural	9.9 (5.9 to 16.1)	9.6 (5.3 to 16.8)	6.9 (3.9 to 12.1)	6.8 (4.4 to 10.5)	15.5 (6.9 to 31.2)	1.05 (0.81 to 1.37)	0.311

© 2020 Ueda P et al. *JAMA Network Open.*

eTable 16. Trends in Having No Sexual Partner in the Last Year Among Men in Sociodemographic Subgroups

		2000/2002	2004/2006	2008/2010	2012/2014	2016/2018		
		**% (95% CI)**	**% (95% CI)**	**% (95% CI)**	**% (95% CI)**	**% (95% CI)**	**Age-adjusted OR for study period**	*p* for interaction
Race	White	10.9 (8.6 to 13.7)	9.6 (7.6 to 12)	8.6 (6.4 to 11.6)	11.7 (8.6 to 15.7)	16.2 (12.4 to 20.9)	1.11 (1.01 to 1.23)	
	Black	6.3 (3.2 to 12.1)	5.5 (2.1 to 13.6)	6.6 (2.7 to 15.1)	19.1 (11.8 to 29.5)	12.5 (6.9 to 21.6)	1.3 (1.05 to 1.62)	
	Other	8.9 (4.4 to 16.9)	15 (7.9 to 26.5)	18 (10.3 to 29.5)	16.7 (11.1 to 24.4)	23 (13.3 to 36.9)	1.3 (1.04 to 1.61)	0.203
Sexual orientation	Heterosexual	-	-	8.9 (6.9 to 11.5)	13 (10.2 to 16.4)	16.5 (12.9 to 21)	1.4 (1.15 to 1.71)	
	Gay/lesbian/bisexual	-	-	16.6 (6 to 38.4)	23.3 (12.6 to 38.9)	4.7 (0.6 to 29.1)	0.52 (0.26 to 1.03)	0.009
Religion	None	13 (8.5 to 19.2)	10.2 (6.2 to 16.4)	9.3 (6.1 to 13.9)	17.4 (11.9 to 24.8)	12.8 (8.8 to 18.3)	1.08 (0.93 to 1.25)	
	Christian	9.5 (7.4 to 12.1)	9.2 (7 to 11.9)	9.3 (6.8 to 12.6)	9.5 (7 to 12.8)	17.2 (12.5 to 23.2)	1.14 (1.02 to 1.28)	
	Non-Christian	7.8 (3.6 to 15.9)	18.2 (9.1 to 33)	17 (6.8 to 36.5)	34.8 (21.1 to 51.5)	25.1 (11.1 to 47.6)	1.36 (1.07 to 1.74)	0.198
Education	High school or less	10.9 (8.5 to 13.8)	11.4 (8.4 to 15.2)	9.6 (6.9 to 13.1)	15.5 (11.9 to 20)	18.1 (13.8 to 23.5)	1.15 (1.04 to 1.27)	
	College or more	7.9 (5.4 to 11.4)	7.9 (5.4 to 11.3)	9.7 (6.3 to 14.7)	9.8 (6.9 to 13.8)	13 (8.5 to 19.4)	1.16 (1.01 to 1.34)	0.92
Employment	Fulltime	6.6 (5.1 to 8.6)	7.4 (5.6 to 9.8)	7.7 (5.6 to 10.6)	6.8 (4.8 to 9.6)	11.7 (8.2 to 16.5)	1.13 (1.01 to 1.27)	
	Part-time	24.2 (16 to 34.7)	8.1 (3.5 to 17.3)	12.6 (6.7 to 22.5)	24.1 (14.8 to 36.7)	22.8 (13.3 to 36.2)	1.08 (0.87 to 1.34)	
	Student	19.8 (9.9 to 35.7)	32.2 (22.4 to 43.8)	24.5 (13.8 to 39.8)	35.8 (24.2 to 49.5)	26.7 (15.4 to 42.2)	1.09 (0.89 to 1.34)	
	Not working	14.7 (7.8 to 26.1)	11 (6.3 to 18.7)	8.7 (4.5 to 16.3)	20.4 (12.5 to 31.4)	27.3 (17.1 to 40.5)	1.25 (0.98 to 1.61)	0.726
Married	No	16.2 (13.1 to 19.8)	16.5 (13.3 to 20.3)	14.2 (11.2 to 17.9)	20.6 (16.4 to 25.4)	24.9 (19.8 to 30.8)	1.14 (1.05 to 1.25)	
	Yes	2.1 (1.1 to 3.9)	1.7 (0.7 to 4.1)	2.9 (1.4 to 5.9)	2.9 (1.5 to 5.7)	1.1 (0.3 to 3.4)	0.99 (0.8 to 1.24)	0.254
Region	Northeast	11.8 (7.9 to 17.2)	9.7 (5.8 to 15.7)	10.1 (5.2 to 18.6)	14.5 (9.7 to 21.2)	24.6 (15.2 to 37.3)	1.22 (1.01 to 1.47)	
	Midwest	13.8 (9.6 to 19.3)	9.8 (6.5 to 14.5)	9.9 (4.7 to 19.8)	11.3 (6.3 to 19.6)	14.1 (8.2 to 23.4)	1.02 (0.86 to 1.21)	
	South	6 (3.7 to 9.5)	9.7 (6.5 to 14.3)	7.6 (5 to 11.4)	12.7 (9 to 17.6)	15.5 (9.7 to 23.7)	1.27 (1.09 to 1.49)	
	West	10 (6 to 16.1)	10.7 (6.9 to 16.3)	11.8 (8.2 to 16.8)	15.9 (9.9 to 24.5)	14.8 (9.4 to 22.5)	1.13 (0.96 to 1.33)	0.826
Residence	City >250,000.	8.2 (4.8 to 13.9)	9.1 (5.6 to 14.3)	9.1 (5.1 to 15.7)	15.2 (9.5 to 23.4)	20.4 (13.8 to 29.1)	1.34 (1.12 to 1.59)	
	City 50-250,000	6.7 (3.5 to 12.3)	12.8 (7.7 to 20.7)	9.8 (6 to 15.5)	14.2 (9 to 21.6)	13.2 (7.3 to 22.6)	1.14 (0.95 to 1.38)	
	Suburbs/small city	11.8 (9.1 to 15.2)	9.2 (6.2 to 13.4)	10.8 (7.3 to 15.8)	14.7 (10.9 to 19.5)	17.1 (12.2 to 23.5)	1.14 (1.01 to 1.28)	
	Rural	10.4 (6.3 to 16.6)	9.6 (5.4 to 16.5)	6.4 (3.6 to 11.1)	5.9 (3.5 to 9.7)	11.8 (5 to 25.4)	0.95 (0.74 to 1.24)	0.06

© 2020 Ueda P et al. *JAMA Network Open.*

eTable 17. Trends in Weekly Sex in Last Year Among Men in Sociodemographic Subgroups

		2000/2002	2004/2006	2008/2010	2012/2014	2016/2018		
		**% (95% CI)**	**% (95% CI)**	**% (95% CI)**	**% (95% CI)**	**% (95% CI)**	**Age-adjusted OR for study period**	*p* for interaction
Race	White	58.7 (54.9 to 62.4)	58.5 (54.5 to 62.5)	57.4 (52.8 to 61.8)	54.7 (49.9 to 59.4)	46.5 (41.5 to 51.6)	0.9 (0.85 to 0.95)	
	Black	70.1 (61.7 to 77.4)	56.6 (44.1 to 68.4)	63 (51 to 73.6)	52.2 (42.8 to 61.5)	46 (34.8 to 57.5)	0.81 (0.7 to 0.92)	
	Other	62 (51 to 71.9)	55.7 (44.3 to 66.4)	53.9 (41.9 to 65.4)	49.1 (40.2 to 58)	48.8 (34.4 to 63.4)	0.86 (0.73 to 1)	0.386
Sexual orientation	Heterosexual	-	-	57.8 (53.6 to 61.9)	54.2 (50.4 to 58.1)	47.2 (42.4 to 51.9)	0.81 (0.72 to 0.92)	
	Gay/lesbian/bisexual	-	-	57.6 (35.4 to 77.1)	36.4 (25 to 49.5)	44.9 (25.7 to 65.7)	0.86 (0.45 to 1.65)	0.88
Religion	None	57.6 (50.8 to 64.1)	62.2 (54.4 to 69.4)	63.8 (56.5 to 70.6)	51.7 (44.7 to 58.7)	50.5 (43.8 to 57.2)	0.9 (0.83 to 0.99)	
	Christian	62.1 (58.2 to 65.8)	57.1 (52.6 to 61.4)	56.2 (51.6 to 60.8)	56.3 (51.3 to 61.2)	44.8 (38.9 to 50.8)	0.88 (0.82 to 0.93)	
	Non-Christian	52.6 (40.6 to 64.4)	51.3 (35.9 to 66.5)	38.2 (23.3 to 55.7)	32.1 (19.8 to 47.5)	43.1 (25.9 to 62.1)	0.86 (0.7 to 1.05)	0.586
Education	High school or less	62 (57.8 to 66.1)	57.3 (52.5 to 61.9)	60.7 (55.8 to 65.4)	54.9 (50.3 to 59.5)	45.7 (40.1 to 51.4)	0.88 (0.83 to 0.94)	
	College or more	56.5 (51.5 to 61.3)	58.9 (53 to 64.5)	50.7 (43.7 to 57.6)	50.3 (44.5 to 56)	48.8 (41.7 to 55.9)	0.91 (0.84 to 0.98)	0.599
Employment	Fulltime	64.7 (61.3 to 68)	62.8 (58.7 to 66.7)	61.1 (56.2 to 65.9)	58.5 (53.9 to 63)	49 (44 to 54)	0.87 (0.82 to 0.92)	
	Part-time	45.4 (34.1 to 57.2)	41 (29.4 to 53.8)	50.8 (38.2 to 63.4)	45.3 (32.3 to 59)	38.8 (27 to 52.2)	0.96 (0.81 to 1.13)	
	Student	39.7 (28.3 to 52.3)	25.5 (16.4 to 37.2)	42.4 (28.6 to 57.5)	36.9 (25.3 to 50.3)	35.7 (23 to 50.8)	1.03 (0.86 to 1.23)	
	Not working	56.8 (44.7 to 68.2)	63.1 (49.8 to 74.7)	55.8 (44.8 to 66.3)	49.1 (39 to 59.2)	51.6 (39.3 to 63.6)	0.91 (0.78 to 1.07)	0.153
Married	No	52.5 (48.3 to 56.6)	49.9 (44.7 to 55)	51.8 (46 to 57.6)	48.5 (43.6 to 53.5)	40.9 (35.6 to 46.4)	0.91 (0.86 to 0.97)	
	Yes	71.1 (66.3 to 75.5)	68.4 (63.2 to 73.2)	66.2 (60.2 to 71.7)	60.8 (54.2 to 67.1)	57.7 (51.3 to 63.9)	0.86 (0.79 to 0.93)	0.262
Region	Northeast	59.7 (51.5 to 67.4)	59.6 (49.2 to 69.2)	56.2 (43.4 to 68.3)	52.5 (43 to 61.9)	44.5 (33.7 to 55.7)	0.87 (0.77 to 0.99)	
	Midwest	55.2 (48.2 to 61.9)	58.7 (50.5 to 66.5)	48.8 (36.9 to 60.9)	49.5 (42.9 to 56.2)	49.4 (41.9 to 57)	0.92 (0.84 to 1.01)	
	South	64.4 (58.7 to 69.8)	58.4 (52.9 to 63.8)	62.7 (56.5 to 68.6)	59.6 (54.8 to 64.2)	45.4 (37.7 to 53.3)	0.87 (0.8 to 0.95)	
	West	62.1 (56.1 to 67.7)	55.3 (46.8 to 63.5)	57.2 (50.2 to 63.9)	49.5 (40.2 to 58.9)	47.2 (38.8 to 55.7)	0.88 (0.8 to 0.97)	0.667
Residence	City >250,000.	56.7 (49.2 to 63.8)	55.8 (46.8 to 64.5)	55.2 (46.7 to 63.4)	55.9 (47 to 64.4)	40.6 (32.3 to 49.5)	0.88 (0.8 to 0.98)	
	City 50-250,000	60 (52 to 67.4)	53.9 (46.1 to 61.5)	54.8 (46.9 to 62.5)	53.6 (45.9 to 61.2)	43.9 (35.9 to 52.2)	0.89 (0.8 to 0.98)	
	Suburbs/small city	61.2 (56.5 to 65.7)	58.1 (52.5 to 63.4)	58.5 (52.1 to 64.7)	48.1 (42.3 to 53.9)	52 (45.8 to 58.2)	0.89 (0.83 to 0.95)	
	Rural	62.6 (53.2 to 71.1)	64.8 (55.5 to 73.1)	64.4 (54.8 to 72.8)	67.8 (61.6 to 73.4)	43 (29.9 to 57.1)	0.9 (0.78 to 1.03)	0.956

© 2020 Ueda P et al. *JAMA Network Open.*

eTable 18. Odds ratios of Survey Period for Sexual Inactivity, Weekly Sex, and No Sexual Partners in the Last Year Among Men in Logistic Regression Models, With and Without Adjustment for Sociodemographic Variables

	OR (Age-adjusted model)	OR (Fully adjusted model)
Sexual inactivity[a]	1.17 (1.07 to 1.28)	1.15 (1.05 to 1.27)
Weekly sex[a]	0.89 (0.84 to 0.93)	0.89 (0.85 to 0.94)
No sexual partners[b]	1.14 (1.05 to 1.25)	1.13 (1.03 to 1.23)

Fully adjusted models included the independent variables age, race, religion, education, employment, marital status, region, and area of residence.

a. The analyses included n=4203 men with data available on all variables included in the fully adjusted model. n=88 were excluded due to missing data on any of the variables.

a. The analyses included n=4284 men with data available on all variables included in the fully adjusted model. n=88 were excluded due to missing data on any of the variables.

© 2020 Ueda P et al. *JAMA Network Open.*

eFigure 1. Distribution of Frequency of Sex and Number of Sexual Partners in the Last Year Among US Men and Women Aged 18-44 in the General Social Survey, 2016-2018

Error Bars Represent 95% CI.

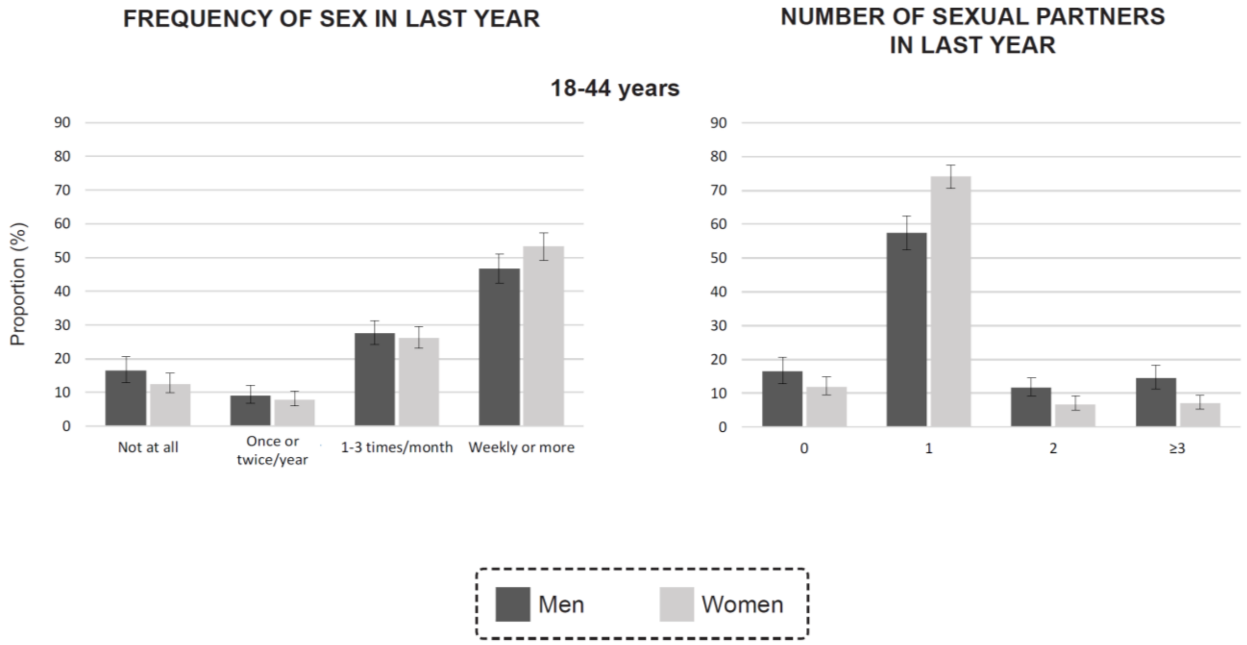

© 2020 Ueda P et al. *JAMA Network Open.*

eFigure 2. The Proportion of Men and Women Who Were Sexually Inactive in the Last Year by Survey Period and Age

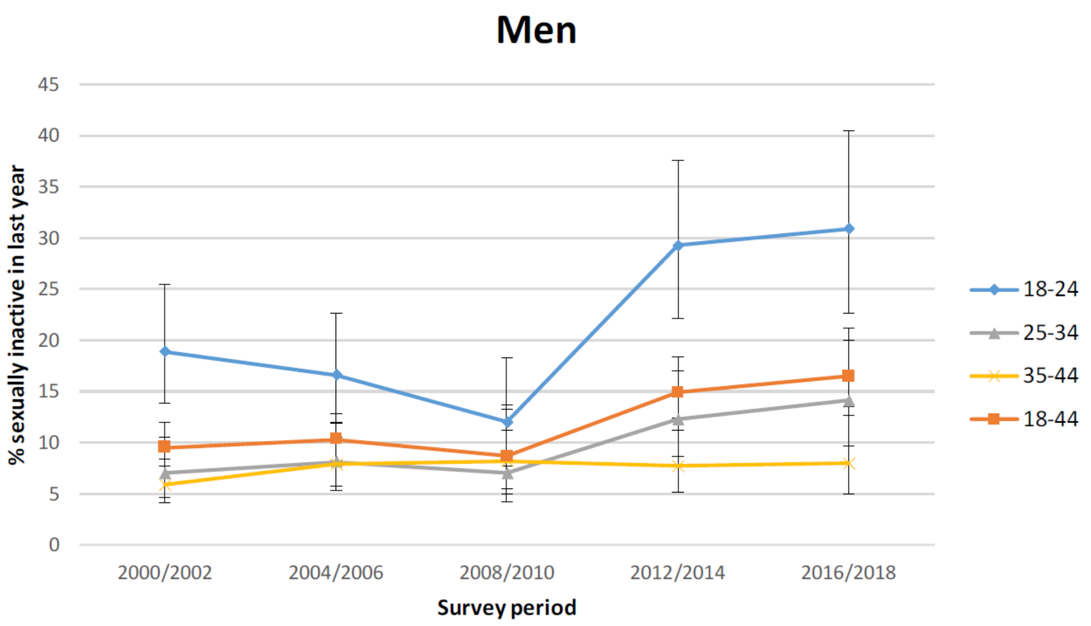

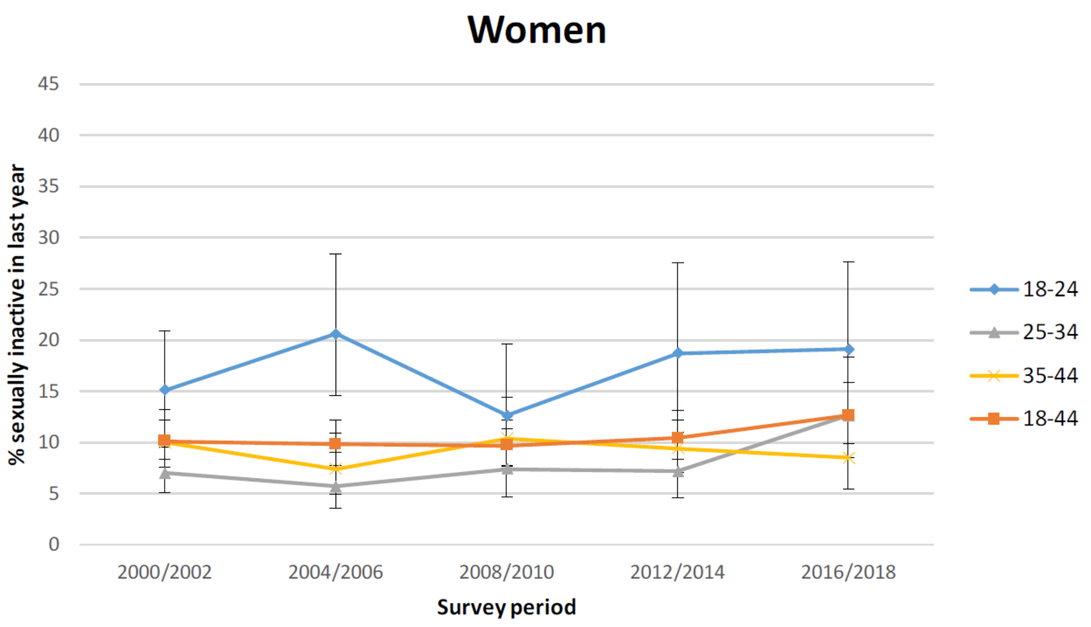

© 2020 Ueda P et al. *JAMA Network Open.*

eFigure 3. Trends in Sexual Frequency and Number of Sexual Partners in the Last Year Among US Men and Women, Aged 18-44 Years, by Marital Status

Error bars represent 95% CI.

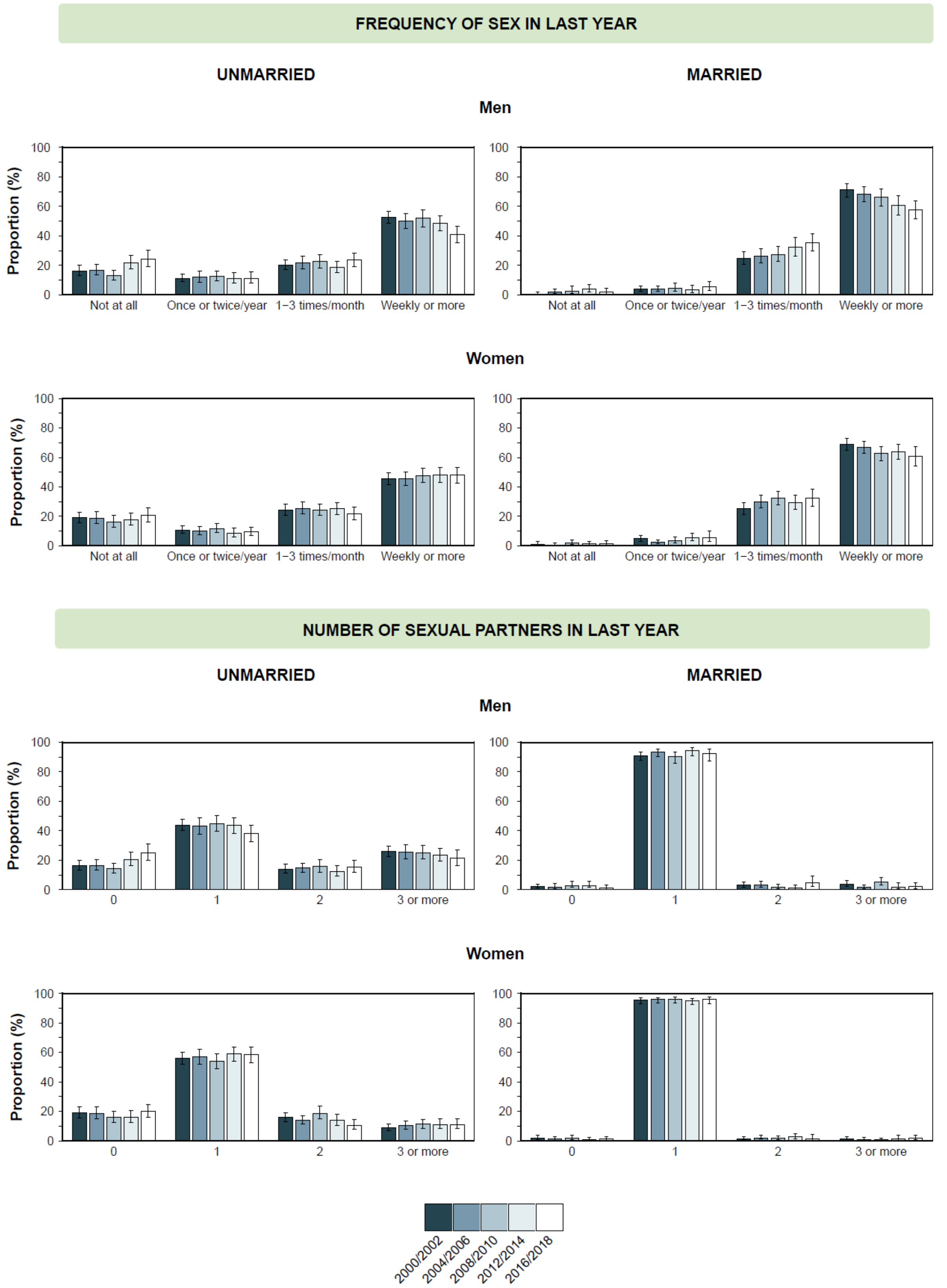

© 2020 Ueda P et al. *JAMA Network Open.*

eFigure 4. Frequency of Sex in Last Year by Survey Year

Error bars represent 95% CI.

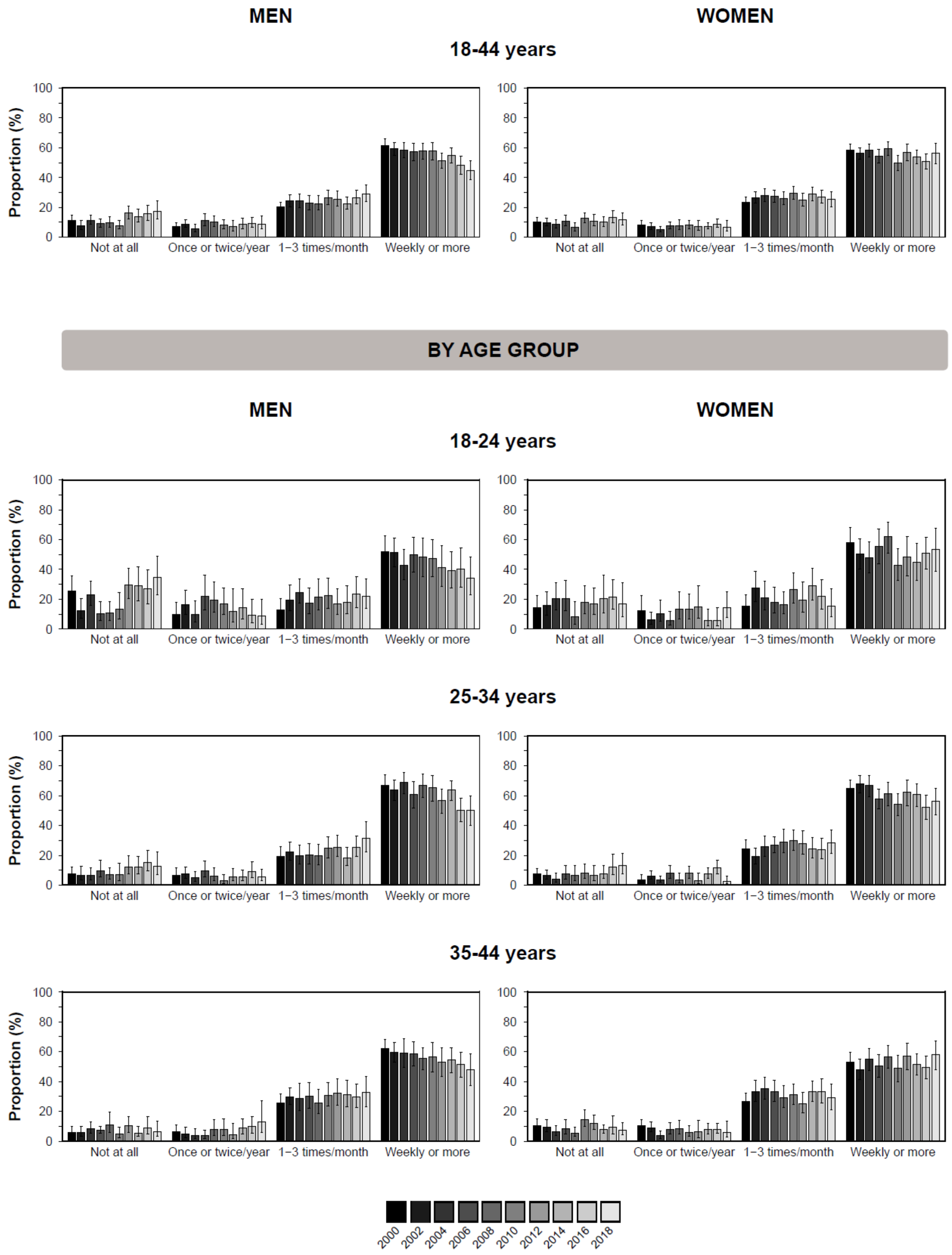

© 2020 Ueda P et al. *JAMA Network Open.*

eFigure 5. Number of Sexual Partners in Last Year by Survey Year

Error Bars Represent 95% CI.

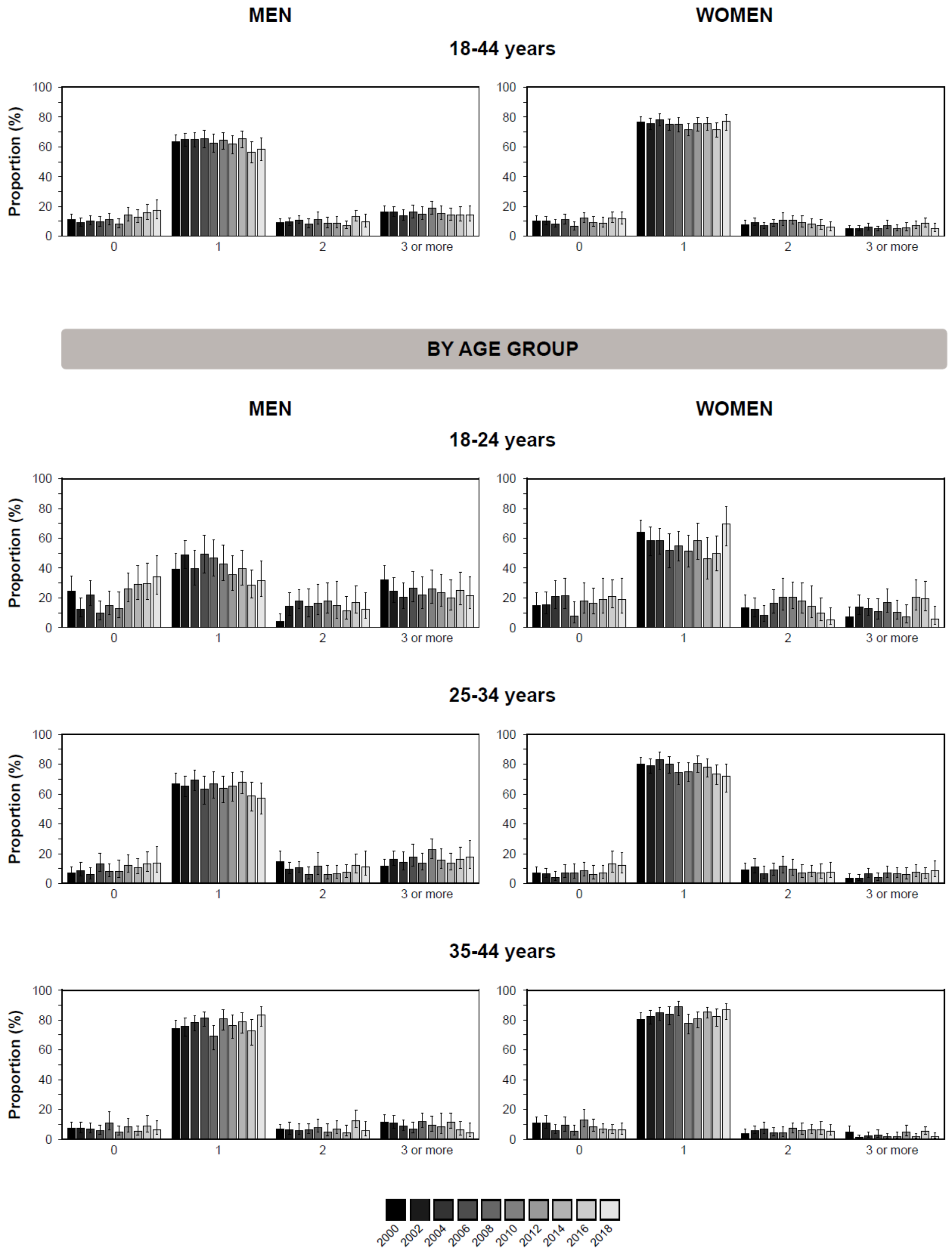

© 2020 Ueda P et al. *JAMA Network Open.*

eFigure 6. Frequency of Sex and the Number of Sexual Partners in the Last Year Among Participants Identifying as Gay, Lesbian, or Bisexual

Error bars represent 95% CI. Data from the General Social Survey, 2008-2018.

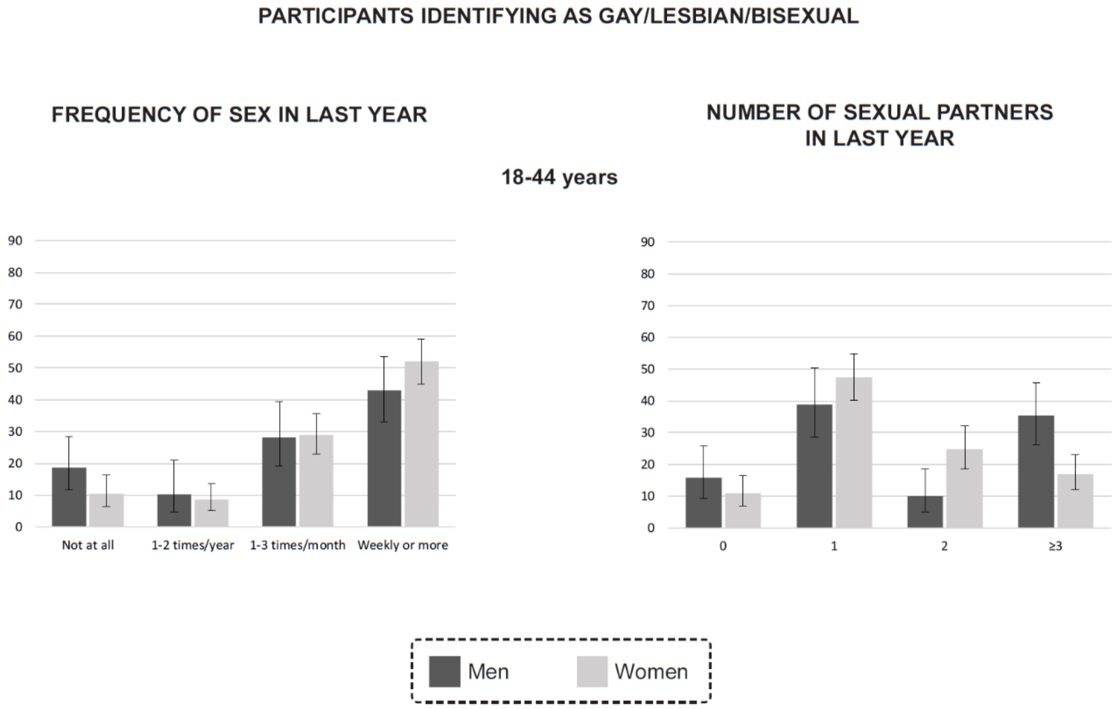

© 2020 Ueda P et al. *JAMA Network Open.*

eReferences

1. Smith TW, Hout M, Marsden P V. General Social Survey, 1972-2016 [Cumulative File] (ICPSR 36797). doi:10.3886/ICPSR36797.v1.

2. General Social Survey. NORC University of Chicago. General Social Survey - Codebook (Appendix A). http://gss.norc.org/Documents/codebook/A.pdf. Accessed 6 Feb 2020.

3. Transparency Initiative - AAPOR. https://www.aapor.org/Transparency_Initiative.htm. Accessed 6 Feb 2020.

4. American Association for Public Opinion Research. Standard Definitions - Final Dispositions of Case Codes and Outcome Rates for Surveys. 2016. https://www.aapor.org/AAPOR_Main/media/publications/Standard-Definitions20169theditionfinal.pdf. Accessed 6 Feb 2020.

5. Wellings K, Palmer MJ, Machiyama K, Slaymaker E. Changes in, and factors associated with, frequency of sex in Britain: evidence from three National Surveys of Sexual Attitudes and Lifestyles (Natsal). BMJ. 2019;:l1525. doi:10.1136/bmj.l1525.

6. Mercer CH, Tanton C, Prah P, Erens B, Sonnenberg P, Clifton S, et al. Changes in sexual attitudes and lifestyles in Britain through the life course and over time: Findings from the National Surveys of Sexual Attitudes and Lifestyles (Natsal). Lancet. 2013;382:1781–94. doi:10.1016/S0140-6736(13)62035-8.

7. Fales MR, Frederick DA, Garcia JR, Gildersleeve KA, Haselton MG, Fisher HE. Mating markets and bargaining hands: Mate preferences for attractiveness and resources in two national U.S. studies. Pers Individ Dif. 2016;88:78–87. doi:10.1016/j.paid.2015.08.041.

8. Hitsch GJ, Hortaçsu A, Ariely D. What makes you click?—Mate preferences in online dating. Quant Mark Econ. 2010;8:393–427. doi:10.1007/s11129-010-9088-6.

9. National Institute of Population and Social Security Research. The Fifteenth Japanese National Fertility Survey in 2015. Marriage Process and Fertility of Married Couples Attitudes toward Marriage and Family among Japanese Singles. Summary of the Survey Results on Married Couples/Singles. 2017. http://www.ipss.go.jp/ps-doukou/j/doukou15/doukou15_gaiyo.asp. Accessed 22 Jun 2018.

10. Buunk BP, Dijkstra P, Fetchenhauer D, Kenrick DT. Age and gender differences in mate selection criteria for various involvement levels. Pers Relatsh. 2002;9:271–8. doi:10.1111/1475-6811.00018.

11. Kim JH, Tam WS, Muennig P. Sociodemographic Correlates of Sexlessness Among American Adults and Associations with Self-Reported Happiness Levels: Evidence from the U.S. General Social Survey. Arch Sex Behav. 2017;46:2403–15. doi:10.1007/s10508-017-0968-7.

12. Lichter DT, Price JP, Swigert JM. Mismatches in the Marriage Market. J Marriage Fam. 2019;:jomf.12603. doi:10.1111/jomf.12603.

13. Ghaznavi C, Sakamoto H, Yoneoka D, Nomura S, Shibuya K, Ueda P. Trends in heterosexual inexperience among young adults in Japan: analysis of national surveys, 1987–2015. BMC Public Health. 2019;19:355. doi:10.1186/s12889-019-6677-5.

© 2020 Ueda P et al. *JAMA Network Open.*

14. Bruch EE, Newman MEJ. Aspirational pursuit of mates in online dating markets. Sci Adv. 2018;4:eaap9815. doi:10.1126/sciadv.aap9815.

15. Rudder C. Dataclysm: Who we are (when we think no one's looking). New York: Random House; 2014.

16. Birger J. Date-onomics. How dating became a lopsided numbers game. Workman Publishing; 2015.

17. Berry BJL, Okulicz-Kozaryn A. An Urban-Rural Happiness Gradient. Urban Geogr. 2011;32:871–83. doi:10.2747/0272-3638.32.6.871.

18. Twenge JM, Sherman RA, Wells BE. Declines in Sexual Frequency among American Adults, 1989–2014. Arch Sex Behav. 2017;46:2389–401. doi:10.1007/s10508-017-0953-1.

19. Ueda P, Mercer CH. Prevalence and types of sexual inactivity in Britain: Analyses of national cross-sectional probability survey data. BMJ Open. 2019;9:1–13.

20. Glick SN, Morris M, Foxman B, Aral SO, Manhart LE, Holmes KK, et al. A comparison of sexual behavior patterns among men who have sex with men and heterosexual men and women. J Acquir Immune Defic Syndr. 2012;60:83–90. doi:10.1097/QAI.0b013e318247925e.

21. Haydon AA, Cheng MM, Herring AH, McRee A-L, Halpern CT. Prevalence and Predictors of Sexual Inexperience in Adulthood. Arch Sex Behav. 2014;43:221–30. doi:10.1007/s10508-013-0164-3.

22. Bodenmann G, Atkins DC, Schär M, Poffet V. The association between daily stress and sexual activity. J Fam Psychol. 2010;24:271–9. doi:10.1037/a0019365.

23. Amichai-Hamburger Y, Etgar S. Intimacy and Smartphone Multitasking—A New Oxymoron? Psychol Rep. 2016;119:826–38. doi:10.1177/0033294116662658.

© 2020 Ueda P et al. *JAMA Network Open.*
